# The Arabidopsis leucine-rich repeat receptor kinase MIK2/LRR-KISS connects cell wall integrity sensing, root growth and response to abiotic and biotic stresses

**DOI:** 10.1371/journal.pgen.1006832

**Published:** 2017-06-12

**Authors:** Dieuwertje Van der Does, Freddy Boutrot, Timo Engelsdorf, Jack Rhodes, Joseph F. McKenna, Samantha Vernhettes, Iko Koevoets, Nico Tintor, Manikandan Veerabagu, Eva Miedes, Cécile Segonzac, Milena Roux, Alice S. Breda, Christian S. Hardtke, Antonio Molina, Martijn Rep, Christa Testerink, Grégory Mouille, Herman Höfte, Thorsten Hamann, Cyril Zipfel

**Affiliations:** 1 The Sainsbury Laboratory, Norwich Research Park, Norwich, United Kingdom; 2 Department of Biology, Norwegian University of Science and Technology, Trondheim, Norway; 3 Department of Life Sciences, Imperial College London, London, United Kingdom; 4 Institut Jean-Pierre Bourgin, INRA, AgroParisTech, CNRS, Université Paris-Saclay, Versailles, France; 5 Department of Plant Cell Biology, University of Amsterdam, Amsterdam, The Netherlands; 6 Department of Phytopathology, University of Amsterdam, Amsterdam, The Netherlands; 7 Centro de Biotecnología y Genómica de Plantas (UPM-INIA), Universidad Politécnica de Madrid (UPM)–Instituto Nacional de Investigación y Tecnología Agraria y Alimentaria (INIA), Campus Montegancedo UPM, Pozuelo de Alarcón (Madrid), Spain; 8 Department of Plant Molecular Biology, University of Lausanne, Biophore Building, Lausanne, Switzerland; University of Massachusetts at Amherst, UNITED STATES

## Abstract

Plants actively perceive and respond to perturbations in their cell walls which arise during growth, biotic and abiotic stresses. However, few components involved in plant cell wall integrity sensing have been described to date. Using a reverse-genetic approach, we identified the *Arabidopsis thaliana* leucine-rich repeat receptor kinase MIK2 as an important regulator of cell wall damage responses triggered upon cellulose biosynthesis inhibition. Indeed, loss-of-function *mik2* alleles are strongly affected in immune marker gene expression, jasmonic acid production and lignin deposition. MIK2 has both overlapping and distinct functions with THE1, a malectin-like receptor kinase previously proposed as cell wall integrity sensor. In addition, *mik2* mutant plants exhibit enhanced leftward root skewing when grown on vertical plates. Notably, natural variation in *MIK2* (also named *LRR-KISS*) has been correlated recently to mild salt stress tolerance, which we could confirm using our insertional alleles. Strikingly, both the increased root skewing and salt stress sensitivity phenotypes observed in the *mik2* mutant are dependent on THE1. Finally, we found that MIK2 is required for resistance to the fungal root pathogen *Fusarium oxysporum*. Together, our data identify MIK2 as a novel component in cell wall integrity sensing and suggest that MIK2 is a nexus linking cell wall integrity sensing to growth and environmental cues.

## Author summary

Plants are constantly exposed to external stresses of biotic and abiotic nature, as well as internal stresses, resulting from growth and mechanical tension. Feedback information about the integrity of the cell wall can enable the plant to perceive such stresses, and respond adequately. Plants are known to perceive signals from their environment through receptor kinases at the plant cell surface. Here, we reveal that the *Arabidopsis thaliana* receptor kinase MIK2 regulates responses to cell wall perturbation. Moreover, we find that MIK2 controls root growth angle, modulates cell wall structure in the root tip, contributes to salt stress tolerance, and is required for resistance against a root-infecting pathogen. Our data suggest that MIK2 is involved in sensing cell wall perturbations in plants, whereby it allows the plant to cope with a diverse range of environmental stresses. These data provide an important step forward in our understanding of the mechanisms plants deploy to sense internal and external danger.

## Introduction

Plant cells are surrounded by a thick cell wall that is composed primarily of complex carbohydrates [1]. The cell wall plays a pivotal role in plants, as it provides the mechanical strength that allows the plant to resist both external and internal (turgor) pressure, protects the cell from biotic and abiotic stresses, and forms the interface between neighbouring cells [1]. The main load-bearing elements of the cell wall are cellulose microfibrils, which are interconnected with a matrix consisting of hemicelluloses, pectins, and a small amount of structural proteins [1]. To allow cell expansion and growth as well as to provide protection against biotic and abiotic stress, the plant requires the ability to adjust the chemical and mechanical properties of the cell wall, for which it requires feedback information about wall integrity. Yeast cells possess an active cell wall integrity (CWI) maintenance mechanism that monitors the status of the cell wall and activates compensatory responses upon damage [2]. Evidence is emerging that plants also have an active CWI sensing mechanism [1, 3–8]. In plants, cell wall damage can be induced in a controlled manner through pharmacological or genetic inhibition of the cellulose synthase complex [1, 3, 5]. Disruption of CWI through inhibition of cellulose biosynthesis results in activation of several stress responses including production of reactive oxygen species [9], jasmonic acid (JA), salicylic acid (SA), and ethylene [10, 11], changes in cell wall composition including lignin deposition [12, 13], callose deposition [13], and alterations in pectin methyl-esterification status [14–16], and finally swollen roots and growth inhibition [17]. Interestingly, these stress responses are reminiscent of the plant’s defence reaction to pathogens and insects [1, 3, 5, 6, 18].

The initiation of the plant’s defence response against pathogens requires perception of pathogen-associated molecular patterns or damage-associated molecular patterns through plasma membrane-localized receptor kinase (RK) proteins [19]. These RK proteins contain an extracellular ligand binding domain, a single-pass transmembrane domain, and an intra-cellular kinase domain [20]. Analogous to their role in pathogen recognition, RKs could be ideal candidates as sensors of CWI, as they allow signal transmission from the external environment to the inside of the cell. In the model plant *Arabidopsis thaliana* (*At*, hereafter referred to as Arabidopsis), the family of RKs contains over 400 members [21]. Several RKs have been identified as putative CWI sensors [1, 4–8, 22], among them the cell surface-localized RK THESEUS1 (THE1) [23]. THE1 was identified in a screen for suppressors of *prc1-1*, a mutant in the cellulose synthase subunit CesA6 [23], and belongs to the malectin-like *Catharanthus roseus* Receptor-Like Kinase 1-like (CrRLK1L) family [4]. While the cellulose-deficient mutant *prc1-1* displays constitutive growth inhibition and lignin deposition, these phenotypes were partially relieved in the *prc1-1 the1-1* double mutant [23]. As *the1-1* does not impact cellulose biosynthesis in *prc1-1* mutant background, it was suggested that THE1 functions as a CWI sensor [23].

The CrRLK1L family contains 17 members in Arabidopsis, and besides THE1, includes FERONIA/SIRENE (FER/SRN), HERCULES1 (HERK1), HERCULES2 (HERK2), ANXUR1 (ANX1), ANXUR2 (ANX2), ERULUS/[CA^2+^]_CYT_-ASSOCIATED PROTEIN KINASE 1 (ERU/ CAP1) and CURVY (CVY1) [4, 6–8]. The extracellular portion of CrRLK1L proteins shows homology to the animal Malectin protein that has putative carbohydrate binding capacity [24]. The above listed CrRLK1L proteins play roles in diverse environmental contexts, possibly linked to CWI sensing [4, 6–8]. THE1, FER and HERK1/2 were found to be required for cell elongation during vegetative growth [25]. FER and ERU have been implicated in polar growth of root hairs [26–28], and CVY1 was found to control leaf cell morphology and actin cytoskeleton organization [29]. Importantly, FER was recently identified as the receptor for the endogenous peptides RAPID ALKALINIZATION FACTOR 1 (RALF1) and RALF23 that control cell elongation inhibition and immune signaling, respectively [27, 30]. Furthermore, FER was identified as a key regulator in mechano-sensing, as *fer* mutant plants show impaired mechanically-induced changes in Ca^2+^ signalling, transcription and growth [31]. FER was initially implicated in pollen tube reception in the female gametophyte. In *fer* mutant ovules, pollen tubes do not burst to release the sperm, but instead continue to grow [32–34]. The related ANX1 and 2 are also involved in pollen tube discharge, yet opposite to *fer* pollen tubes, *anx1/2* pollen tubes burst prematurely [35–37]. Finally, *fer* mutants display enhanced resistance to the powdery mildew *Golovinomyces orontii* [38], and the fungus *Fusarium oxysporum* [39], which may reflect a role of FER in fungal haustorium formation, while *fer* mutants are also affected in flg22-induced signalling and are more susceptible to the bacterium *Pseudomonas syringae* pv. tomato DC3000 [30]. In addition to CrRLK1Ls, another RK subfamily of interest in the context of CWI sensing is the family of wall-associated kinases (WAKs). WAKs can bind pectin [40, 41], and WAK1 is involved in the perception of oligogalacturonides (OGAs) [42], which are breakdown products of pectin that can elicit defence responses [43]. In addition, WAKs have been shown to be required for normal cell elongation [44]. Moreover, leucine-rich repeat receptor kinases (LRR-RKs) have also been associated with CWI sensing [45]. For example, loss-of-function of the LRR-RK-encoding genes *FEI1* and *FEI2* results in hypersensitivity to inhibition of cellulose biosynthesis, high sucrose and high salt, and disrupts anisotropic cell expansion and synthesis of cell wall polymers [45].

However, the CrRLK1L THE1 is so far the only RK that was shown to be required for responses to cellulose biosynthesis inhibition. In this study, we expand our understanding of CWI sensing by identifying the recently characterised LRR-RK MALE DISCOVERER 1-INTERACTING RECEPTOR LIKE KINASE 2/LEUCINE-RICH REPEAT KINASE FAMILY PROTEIN INDUCED BY SALT STRESS (MIK2/LRR-KISS; hereafter referred to as MIK2) [46, 47] as being required for responses to cellulose biosynthesis inhibition. MIK2 shows overlapping as well as distinct functions with THE1 in response to cellulose biosynthesis inhibition. In addition, we find that MIK2 is required for control of normal root growth direction and salt tolerance in a THE1-dependent manner. Moreover, MIK2 plays a role in immunity as it is required for resistance to the fungal root pathogen *Fusarium oxysporum*. We thus propose that MIK2 is involved in CWI sensing and regulates several aspects of growth, as well as responses to abiotic and biotic stresses.

## Results

### The LRR-RK MIK2 is an important regulator of responses triggered by cellulose biosynthesis inhibition

**Fig 1:**
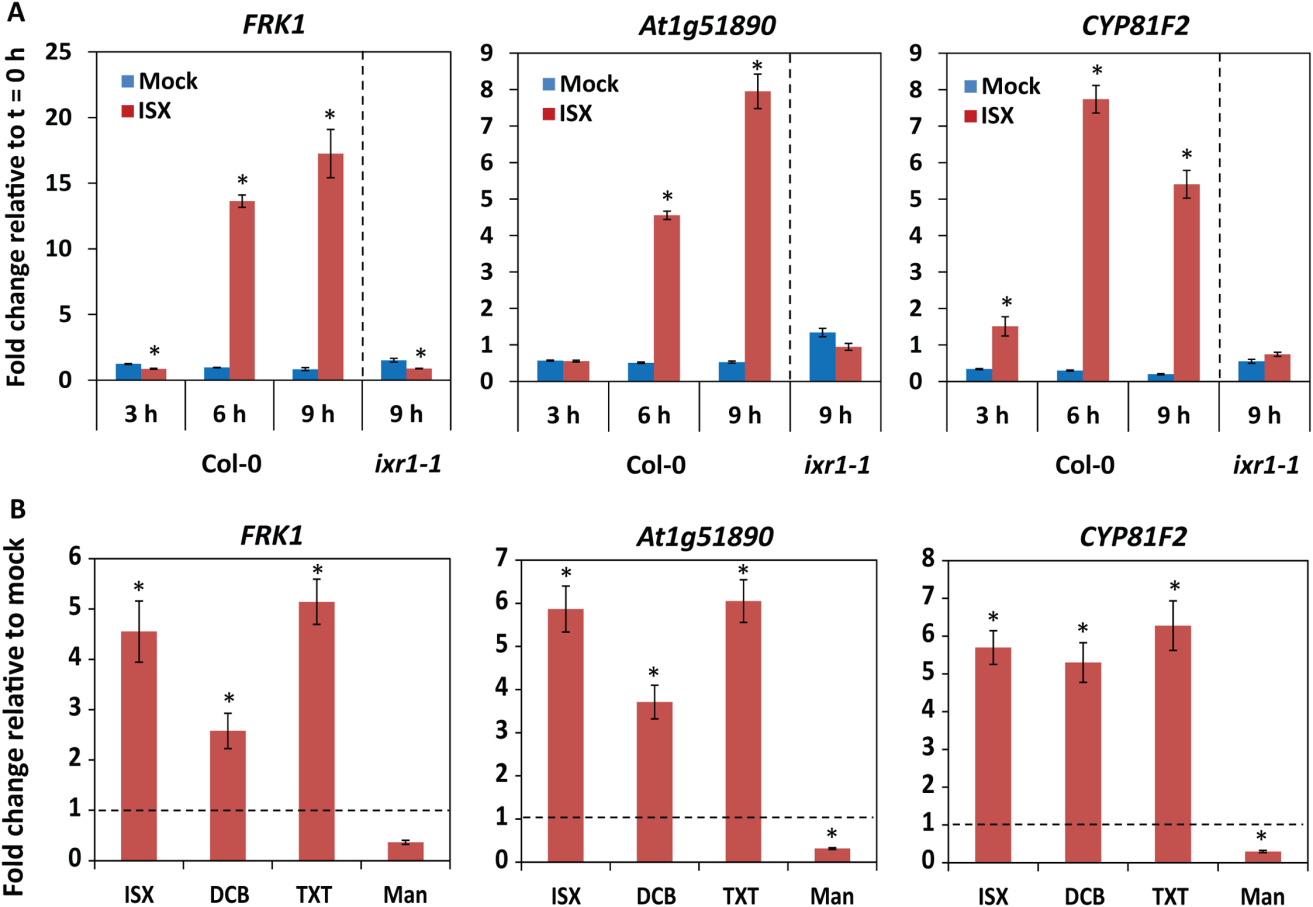
Inhibition of cellulose biosynthesis induces immune marker gene expression. (A,B) Immune marker gene expression in 13-day-old Arabidopsis seedlings determined by qRT-PCR. (A) Seedlings were mock- or ISX-treated (0.6 μM) for the indicated periods. (B) Seedlings were mock treated, or treated with 0.6 μM ISX, 6 μM DCB, 0.4 μM TXT, or 400 mM Mannitol (Man) for 9 h. (A,B) Expression of the immune marker genes *FRK1*, *At1g51890*, and *CYP81F2* was normalized relative to *U-box* expression values. Depicted is the fold change in expression relative to time point t = 0h (A), or relative to mock treatment (B). Error bars represent standard error of three technical replicas. Experiments were repeated at least three times with similar results.

An overlap exists between responses activated upon disruption of CWI and the ones triggered by perception of microbes [9, 48] suggesting that CWI signalling and immune signalling might be part of a general ‘danger’ perception system in which loss of CWI would be sensed as ‘altered self’. Consistently, we observed that treatment with isoxaben (ISX), a chemical widely used to disrupt CWI in a controlled manner via the inhibition of cellulose biosynthesis [1, 3, 13], induced the expression of the genes *FRK1*, *At1g51890* and *CYP81F2* in Arabidopsis, which are commonly used immunity marker genes [49] (Fig 1A). While this increased expression was visible in wild-type Col-0 at 6 and 9 h after treatment, it was absent in the ISX-insensitive mutant *ixr1-1* [50] (Fig 1A). Moreover, treatment with other cellulose biosynthesis inhibitors, such as 2,6-di-chlorobenzonitrile (DCB) [51] and thaxtomin (TXT) [52, 53], also induced expression of the same genes (Fig 1B). Mild hyper-osmotic stress triggered by mannitol treatment did not activate, but rather seemed to repress the expression of these genes (Fig 1B), revealing that the response observed upon treatment with cellulose biosynthesis inhibitors differs from the response to hyper-osmotic stress. Given the load-bearing role of cellulose in plant cell walls, its loss/reduction may lead to mechanical disruption of cell wall and membrane integrity, the release of cell wall components (such as carbohydrates or proteins), or the active production/secretion of endogenous peptides in response to cell wall damage. Such molecules or mechanical signals might then act as triggers for cell surface RKs.

**Fig 2:**
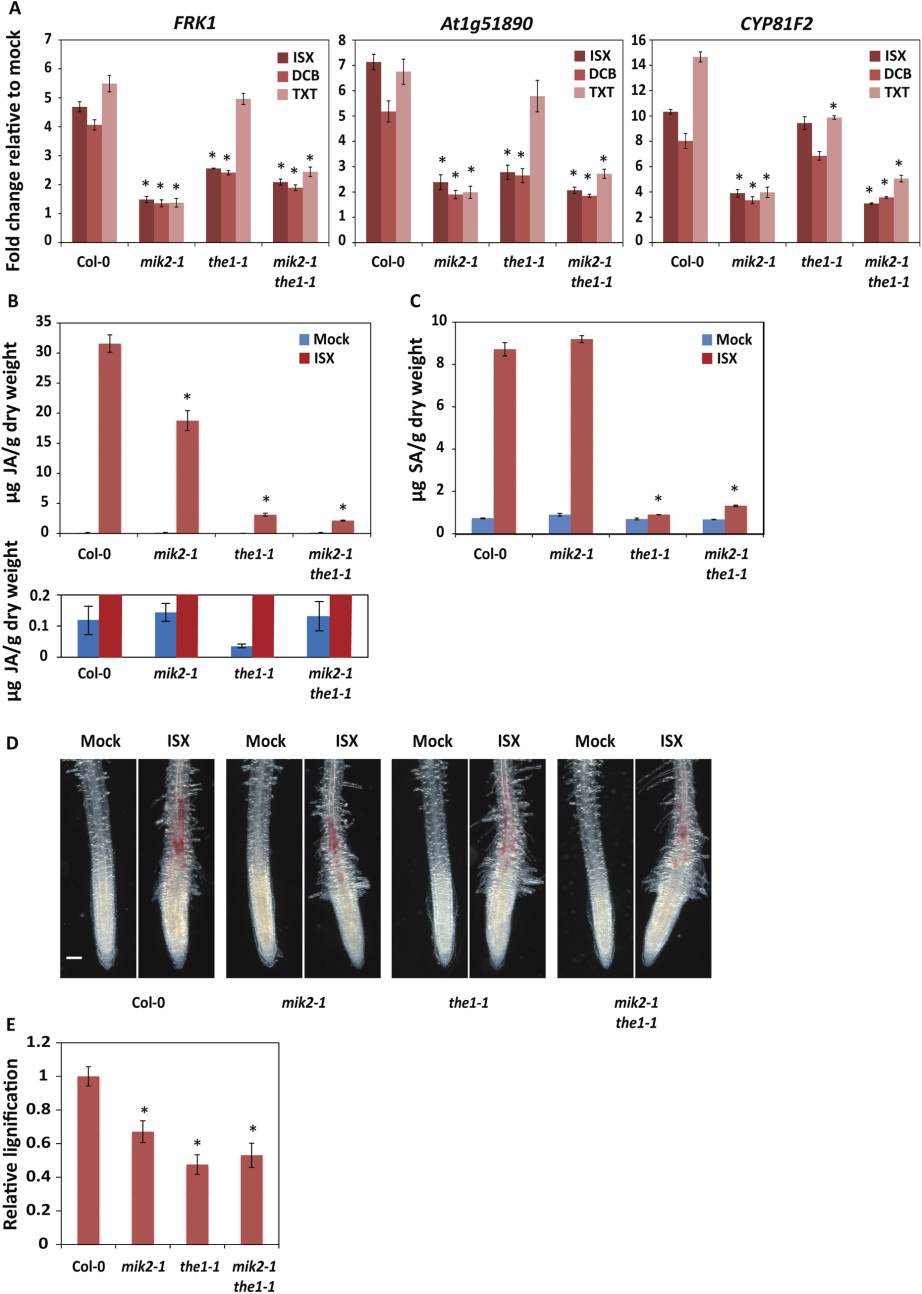
The LRR-RK MIK2 and CrRLK1L THE1 are major regulators of responses triggered by cellulose biosynthesis inhibition. (A) Immune marker gene expression in 13-day-old Arabidopsis seedlings determined by qRT-PCR. Seedlings were mock treated, or treated with 0.6 μM ISX, 6 μM DCB, or 0.4 μM TXT for 9 h. Expression of the immune marker genes *FRK1*, *At1g51890*, and *CYP81F2* was normalized relative to *U-box* expression values. Depicted is the fold change in expression relative to mock treatment. Error bars represent standard error of three technical replicas. (B-E) JA (B) and SA production (C) and lignin-deposition (D,E) in 6-day-old Arabidopsis seedlings, mock treated or treated with 0.6 μM ISX for 7 h (B,C) and 12 h (D,E). Error bars represent standard error of n = 4 (B,C) or n = 20 (E) biological replicas. (B) The upper and lower panel display the same data, yet in the lower panel, the y-axis has been adjusted to visualize the JA levels in mock-treated samples. (D) The size bar represents 100 μm. (A-E) Asterisks indicate a statistically significant difference relative to Col-0, as determined by a two-tailed Student’s *T*-test (*p* < 0.05). Experiments were repeated at least three times with similar results.

To test this hypothesis, we sought to identify RKs that are required for ISX-induced responses and may therefore represent potential components involved in CWI sensing. Towards this end we tested the ISX response of Arabidopsis T-DNA mutants available in our laboratory with insertions in RK-encoding genes. As a result, we identified two independent homozygous insertion alleles in the gene *At4g08850* that displayed reduced ISX-induced immune marker gene expression (Figs 2A and S1A–S1C). This gene encodes a LRR-RK recently characterized as MIK2/LRR-KISS [46, 47]. We found that *mik2-1* was also compromised in DCB- and TXT-induced gene expression (Fig 2A). In addition, *mik2-1* was tested for the previously reported ISX-induced JA and SA accumulation, as well as lignin deposition [3, 9], and the mutant was found to be impaired in ISX-induced JA accumulation and lignin deposition, but not in ISX-induced SA accumulation (Fig 2B–2E). Together, these data demonstrate that MIK2 is an important regulator of responses triggered by cellulose biosynthesis inhibition.

MIK2 contains an extracellular domain consisting of 24 LRRs, a single-pass transmembrane domain, and an intracellular kinase domain (S1D Fig). In accordance with its predicted subcellular localization, MIK2-GFP localized to the plasma membrane (S1E Fig).

MIK2 is part of the sub-family XIIb of LRR-RKs [54, 55] and has a close homolog (60% amino acid identity), At1g35710, that we named MIK2-LIKE (S2A Fig). When compared with LRR-RKs encoded by the rice, tomato, poplar, grapevine and soybean genomes, AtMIK2 is more similar to AtMIK2-LIKE than to any of the rice, tomato, poplar, grapevine or soybean sequences [55–58]. On the other hand, in the *Brassicaceae* species *Arabidopsis lyrata* and *Brassica rapa*, MIK2 and MIK2-LIKE paralogs clearly exist (S2A Fig). *AtMIK2* and *AtMIK2-LIKE* are expressed throughout the plant, in young as well as in mature tissues (S3 Fig). To investigate the potential redundant role of MIK2-LIKE in responses to cellulose biosynthesis inhibition, two T-DNA insertion alleles for *MIK2-LIKE* (*mik2-like-1 and mik2-like-2;* S2B and S2C Fig), and *mik2-1 mik2-like-1* and *mik2-1 mik2-like-2* double mutants were tested for ISX-induced responses. Unlike *mik2-1*, *mik2-like-1* was not impaired in ISX-induced gene expression, JA accumulation or lignin deposition (S2D–S2F Fig). The *mik2-1 mik2-like-1* and *mik2-1 mik2-like-2* double mutants showed a phenotype similar to the *mik2-1* single mutant (S2D–S2F Fig). Thus, despite their close homology, our data suggest that MIK2-LIKE does not fulfil the same function as MIK2 in responses to cellulose biosynthesis inhibition.

### The LRR-RK MIK2 and CrRLK1L THE1 are major regulators of responses to cellulose biosynthesis inhibition

A prominent CWI sensor candidate is the CrRLK1L THE1, which is required for cellulose biosynthesis inhibition responses in *prc1-1*, a mutant in the cellulose synthase subunit CesA6 [23]. Like MIK2, THE1 is expressed throughout the plant, in young as well as in mature tissues (S3 Fig). We tested if MIK2 and THE1 play similar roles in responses to cellulose biosynthesis inhibition. We found that both *mik2-1* and *the1-1*, as well as the double-mutant *mik2-1 the-1* were impaired in the ISX-induced expression of the immune marker genes *FRK1* and *At1g51890* (Fig 2A). However, while *mik2-1* and *mik2-1 the1-1* were also impaired in the ISX-induced expression of *CYP81F2*, *the1-1* was not (Fig 2A). Interestingly, immune marker gene expression in response to DCB was also compromised in *mik2-1*, *the1-1*, and *mik2-1 the1-1* (Fig 2A). In contrast, immune marker gene expression in response to TXT was only impaired in *mik2-1* and *mik2-1 the-1*, but not in *the1-1* (Fig 2A), suggesting that MIK2 and THE1 might function in the activation of responses to cellulose biosynthesis inhibition through different mechanisms. More in depth knowledge on the difference between ISX-, and TXT-mode-of-action will however be required to gain further insight in the different mechanisms by which MIK2 and THE1 might operate.

ISX-induced JA accumulation was more strongly attenuated in *the1-1* and *mik2-1 the1-1* than in the *mik2-1* single mutant (Fig 2B). ISX-induced SA accumulation was also impaired in *the1-1* and *mik2-1 the1-1*, but not in *mik2-1* (Fig 2C). ISX-induced lignin deposition was impaired to a similar level in *mik2-1*, *the1-1*, and *mik2-1 the1-1* (Fig 2D and 2E). However, unlike THE1, MIK2 is not required for the cellulose biosynthesis inhibition response in the CesA6 mutant *prc1-1*, as loss-of-function of *MIK2* did not rescue the shortened dark-grown hypocotyl phenotype in *prc1-1* plants, while loss-of-function of *THE1* partially did (S4 Fig).

In addition to the above described responses, ISX was previously shown to induce rapid internalization of the cellulose synthase complex and accumulation of the complex in microtubule-associated cellulose synthase compartments (MASCs) in the cell cortex [59–61]. Neither loss-of-function of *MIK2* nor of *THE1* interfered with ISX-induced GFP-CESA3 internalization (S5A–S5C Fig), indicating that MIK2 and THE1 must function either downstream, or independent of cellulose synthase complex internalization.

In all assays, the *mik2-1 the1-1* double mutant displayed the same phenotype as either one of the *mik2-1* or *the1-1* single mutants (Fig 2A–2E), demonstrating that loss-of-function of both *MIK2* and *THE1* does not have an additive effect. From a classical genetics point-of-view this would suggest that the two RKs could function in the same pathway; however, clear differences exist in amplitude as well as type of responses that MIK2 and THE1 regulate (Fig 2A–2E; S4 Fig), indicating that they might also regulate different aspects of the CWI maintenance response.

### MIK2 controls root angle in a THE1- and cellulose synthase-dependent manner

**Fig 3:**
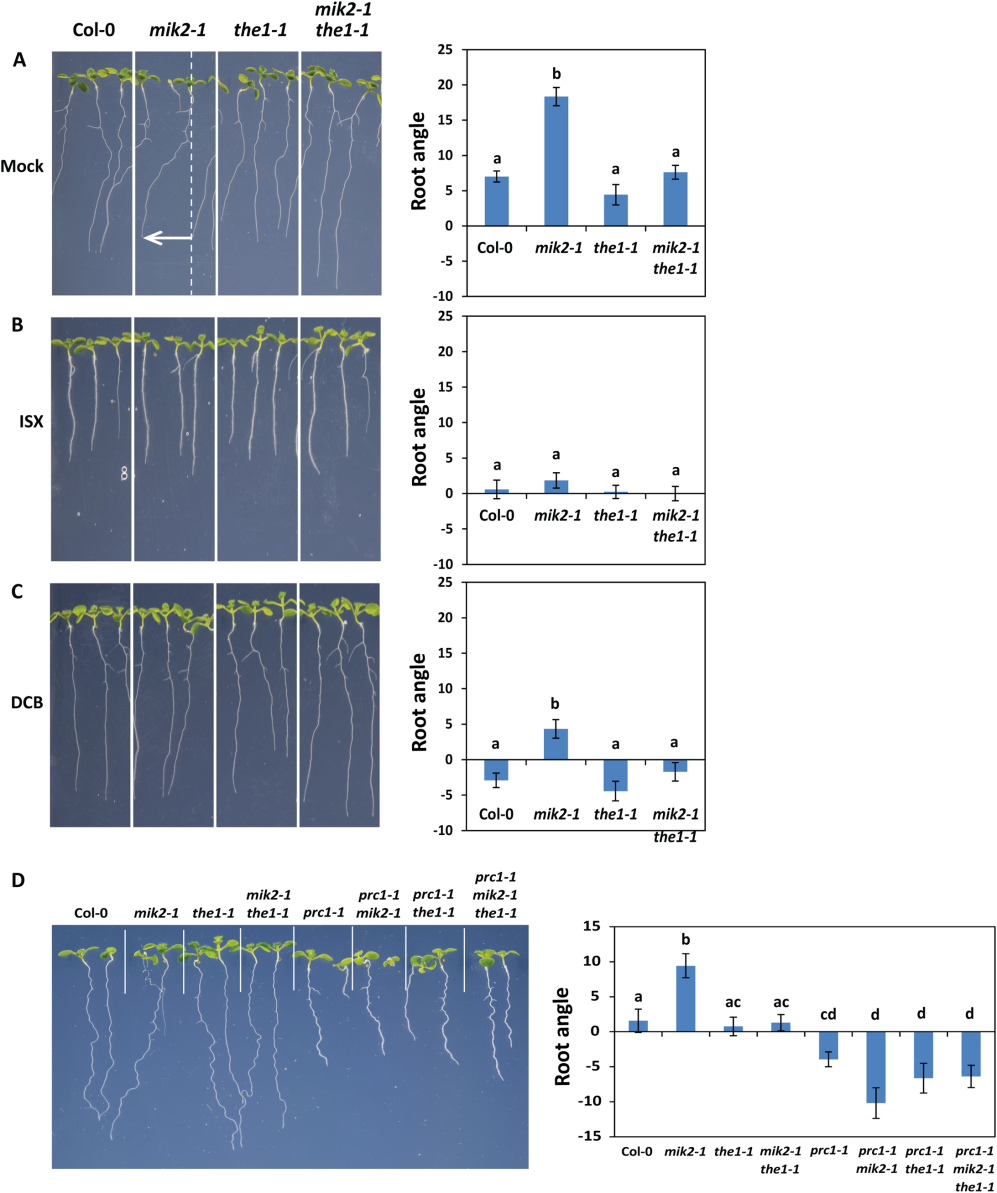
MIK2 controls root angle in a THE1- and cellulose synthase complex-dependent manner. (A-D) Nine-day-old Arabidopsis seedlings grown in an upright position (under a 10° angle relative to the direction of gravity) on MS agar medium with 1% sucrose. Pictures were taken from the front of the plate. (A-C) The growth medium contained DMSO (mock) (A), 2 nM ISX (B), or 25 μM DCB (C). (A) The white arrow indicates skewing of *mik2-1* roots relative to the vertical growth axis. (A-D) Root angle was quantified; a positive value indicates skewing to the left, while a negative value indicates skewing to the right. Error bars represent standard error of n = 15 biological replicas. Different letters indicate statistically significant differences between genotypes (ANOVA and Holm-Sidak test (*p* < 0.05)). The experiments were repeated at least three times with similar results.

It is hypothesized that proper CWI sensing is important for optimal plant growth or development. Interestingly, when grown vertically on MS agar plates, *mik2-1* and *mik2-2* plants displayed left-ward root skewing, while *the1-1* and *the1-4* did not (Fig 3A, S1F Fig, S6A Fig). This effect was previously observed in certain Arabidopsis ecotypes, but is minimal in Col-0 [62]. Surprisingly, this effect was abolished in the *mik2-1 the1-1* double mutant (Fig 3A). Furthermore, we observed that the presence of ISX or DCB in the growth medium impaired root skewing in *mik2-1* (Fig 3B and 3C). The root skewing phenotype of *mik2-1* was also attenuated in the *prc1-1* genetic background (Fig 3D). Thus, these results indicate that MIK2 controls root angle in a THE1- and cellulose synthase-dependent manner.

Although MIK2-LIKE did not fulfil the same function as MIK2 in responses to cellulose biosynthesis inhibition (S2D–S2F Fig), *mik2-like-1* and *mik2-like-2* displayed a trend towards enhanced root skewing (S2G Fig). However, the enhanced root skewing was only found to be statistically significant in 3 out of 6 experiments. Thus, MIK2-LIKE might contribute to the control of root growth angle, yet not to the same extent as MIK2. Surprisingly, the *mik2-1 mik2-like-1* and *mik2-1 mik2-like-2* double mutants displayed a trend towards enhanced root skewing similar to *mik2-like-1* and *mik2-like-2* single mutants, yet reduced compared to the *mik2-1* single mutant. Future work is needed to unravel the genetic relatedness between *MIK2* and *MIK2-LIKE* with respect to control of root growth angle (S2G Fig).

To analyse the potential mechanism underlying the root skewing phenotype of *mik2* mutants, we investigated if roots of *mik2-1* mutants are affected in cellulose microfibril orientation or cell wall structure. Root tips of *mik2-1*, *the1-1*, and *mik2-1 the1-1*, did not display altered cellulose microfibril orientation compared to Col-0 (S7A Fig). Fourier-transform infrared (FT-IR) spectroscopy revealed small differences in the cell wall structure in the root tip of *mik2-1* plants compared to Col-0 (S7B and S7C Fig). The cell wall structure in the root tips of *the1-1* plants was also significantly different from Col-0, yet showed absorption spectra different from *mik2-1* (S7B and S7C Fig), suggesting distinct cell wall modifications. The absorption spectra in the *mik2-1 the1-1* double mutant followed a pattern that was more similar to *the1-1* than *mik2-1* (S7B and S7C Fig), suggesting that the effect of *the1-1* on the cell wall is dominant over the effect of *mik2-1*. Root tip morphology was comparable between *mik2-1* and *the1-1* single mutants, and the *mik2-1 the1-1* double mutant (S8 Fig). Thus, the distinct influences of *mik2-1* and *the1-1* on cell wall structure in the root tip might underlie the observed root skewing, or lack thereof, in the *mik2-1* single mutant and the *mik2-1 the-1* double mutant, respectively. However, biochemical analysis of cell walls from whole roots did not reveal any significant changes in cellulose, hemicellulose or pectin content in the single mutants nor in the *mik2-1 the1-1* double mutant (S9 Fig). The observed cell wall defects in *mik2-1* and *the1-1* are therefore suggestive of subtle, local changes in the root tip, which would need to be confirmed in future, more detailed studies.

### MIK2 is required for salt stress tolerance in a THE1-dependent manner

**Fig 4:**
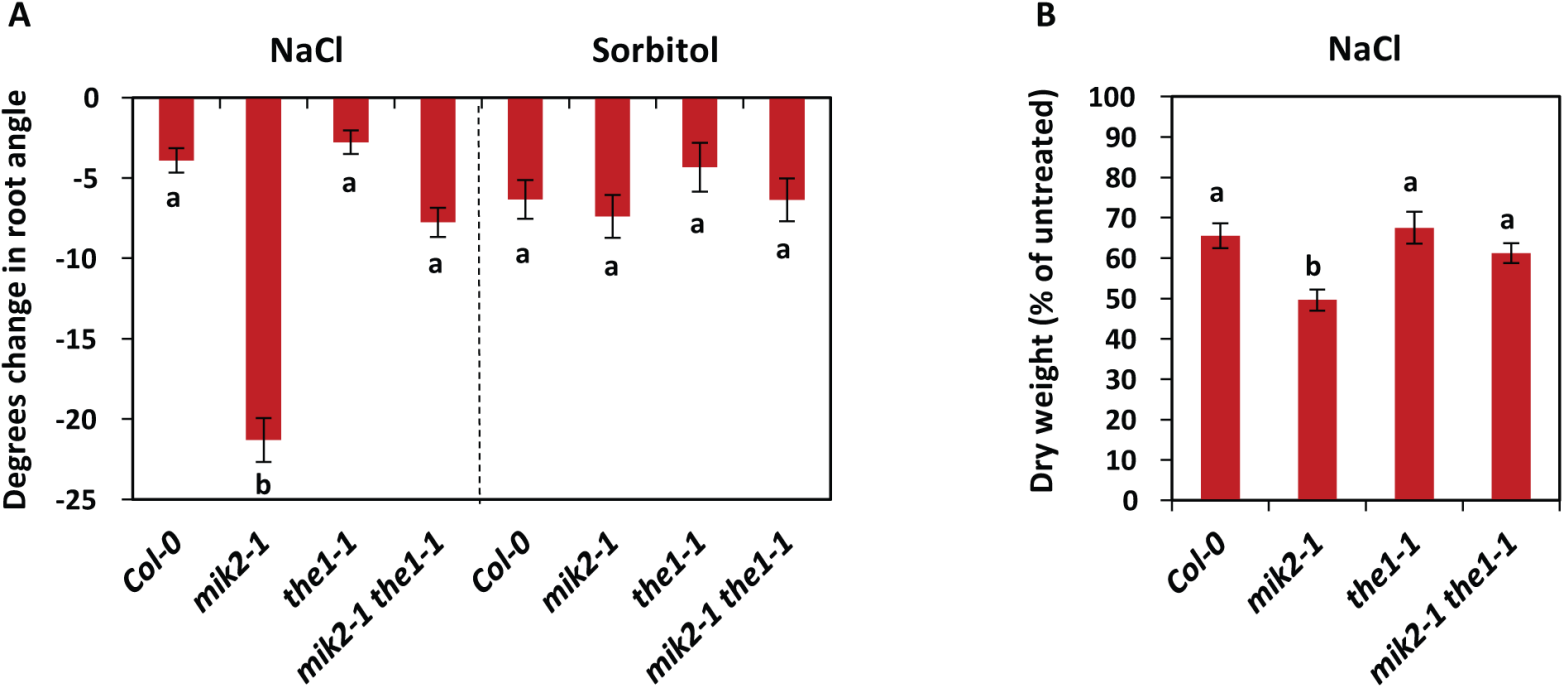
MIK2 is required for salt stress tolerance in a THE1-dependent manner. (A) Ten-day-old Arabidopsis seedlings were grown in an upright position on ½ MS agar medium without sucrose, supplemented with or without 75 mM NaCl or 150 mM sorbitol. Depicted is the change in the angle of the root after NaCl or sorbitol treatment compared to mock treatment; the negative value indicates a change to the right. Error bars represent standard error of n = 20 biological replicas. The experiment was repeated three times with similar results. (B) Dry weight of NaCl-treated plants as percentage of the dry weight of untreated plants. (Absolute dry weight is depicted in S10 Fig). One week after germination, plants were transferred to pots with soil watered from below with or without 75 mM of NaCl in rainwater. After 4 weeks of treatment the rosettes were cut, and dry weight was determined. The experiment was repeated three times with similar results, data were pooled and the average is depicted. Error bars represent the standard error of n = 60 plants. (A,B) Different letters indicate statistically significant differences between genotypes (Kruskal-Wallis ANOVA on ranks followed by Dunn’s multiple comparison procedures (*p* <0.05)).

Recently, natural variation in *MIK2* was found to be linked to shoot growth under salt stress conditions in a study in which it was named *LRR-KISS* [47]. Accessions with *MIK2* expression higher than in Col-0, such as Cen-0, were less sensitive to salt stress, while accessions with *MIK2* expression lower than Col-0, such as HR-5, were more sensitive to it [47]. We were thus curious to test the effects of salt stress on *mik2* insertional mutant plants in the Col-0 background. In line with a previous report [63] we observed that when grown on MS medium containing 75 mM NaCl, Col-0 roots display a mild skewing response to the right, when seen from the front (Fig 4A). In support with the proposed role for MIK2 in salt stress signalling [47], *mik2-1* plants showed a strongly increased right-ward skewing on medium containing 75 mM NaCl, while not on MS medium containing 150 mM sorbitol (Fig 4A). Unlike *mik2-1*, *the1-1* and *the1-4* were not affected in NaCl-induced changes in root growth direction compared to Col-0 (Fig 4A, S6B Fig). The enhanced NaCl-induced right-ward skewing of *mik2-1* roots was abolished in *mik2-1 the1-1* roots (Fig 4A). In support with these observations, we found that NaCl-induced reduction in dry weight of mature plants was enhanced in *mik2-1* compared to wild-type Col-0, while *the1-1* and *the1-4* single and *mik2-1 the1-1* double mutants were not affected in the NaCl-induced decrease of dry weight (Fig 4B, S6C Fig). However, of note is that untreated *mik2-1 the1-1* plants show a slight reduction in dry weight (S10 Fig), suggesting that loss of both MIK2 and THE1 impairs biomass assimilation under basal conditions. Nevertheless, altogether these data show that MIK2 is required for salt stress tolerance in a THE1-dependent manner.

### MIK2 is required for resistance to the fungal root pathogen *Fusarium oxysporum* in a THE1-independent manner

**Fig 5:**
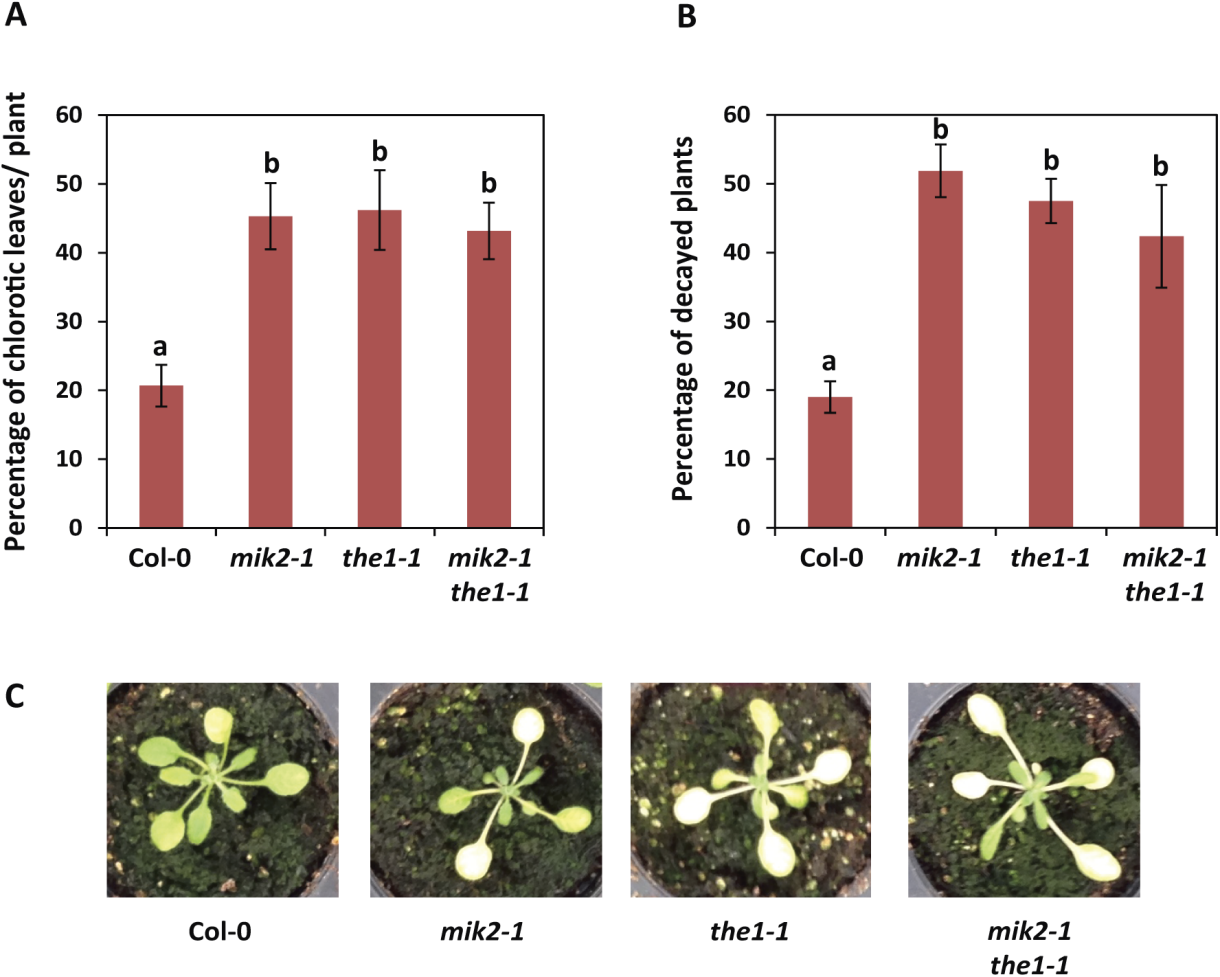
MIK2 is required for resistance to the fungal root pathogen *Fusarium oxysporum* in a THE1-independent manner. (A,B) Percentage of chlorotic leaves per plant (A), and percentage of decayed plants (B) after infection of the roots with *F*. *oxysporum* isolate Fo5176. (A) The percentage of chlorotic leaves per plant was counted 10 days after inoculation with *F*. *oxysporum* spores. (B) The number of decayed plants was counted 3 weeks after inoculation with *F*. *oxysporum* spores. (A,B) The bars represent the average of four independent experiments, each consisting of n = 20–40 plants per genotype. Error bars represent the standard error of n = 4 experiments. Different letters indicate statistically significant differences between genotypes (ANOVA and Holm-Sidak test (*p* < 0.05)). No disease symptoms were observed on mock-inoculated plants for any of the genotypes (n = 10). (C) Representative pictures of the different genotypes in (A) and (B) after *F*. *oxysporum* infection.

Given that cellulose biosynthesis inhibition leads to the induction of MIK2-dependent responses that are similar to those caused upon perception of microbes or wounding, we were curious to test whether MIK2 could play a role in disease resistance. Interestingly, *mik2-1* plants displayed enhanced susceptibility to the root-infecting fungus *Fusarium oxysporum* isolate Fo5176 (Fig 5A–5C). A similar trend was observed in *the1-1* plants, yet was only found to be statistically significant in 4 out of 7 experiments (Fig 5A–5C, S6D and S6E Fig). Mutant *the1-4* plants did not display such an enhanced susceptibility phenotype (Fig 5A–5C, S6D and S6E Fig). The *mik2-1 the1-1* double mutant plants exhibited a phenotype similar to *mik2-1* (Fig 5A–5C). Thus, while MIK2 is required for salt stress tolerance in a THE1-dependent manner, the role of MIK2 in resistance against *Fusarium oxysporum* isolate Fo5176 does not depend on THE1. As we obtained discrepant results with the different alleles for THE1, the exact role of THE1 in resistance to *Fusarium oxysporum* isolate Fo5176 remains to be elucidated.

## Discussion

In this study, we have identified the LRR-RK MIK2 as an important regulator of responses to cellulose biosynthesis inhibition, as evidenced by the impaired gene expression, JA accumulation and lignin deposition triggered by chemical inhibition of cellulose biosynthesis observed in *mik2* mutant plants (Fig 2). This finding suggests a role for MIK2 in transmission of biochemical or physical signals directly derived from the cell wall or indirectly produced/secreted upon cell wall damage triggered upon cellulose biosynthesis inhibition.

In addition, we found that MIK2 plays a role in control of root growth angle (Fig 3). Different Arabidopsis ecotypes are known to display different degrees of left-ward root skewing, yet the molecular basis of root skewing is not well understood [62, 64, 65]. Mechano-sensing, microtubule organization and cell wall composition are suggested to be linked to this phenomenon [62, 64, 65]. Mutants in the CrRLK1L FER are impaired in mechano-sensing and display increased right-handed skewing [31]. The hard agar surface of the growth medium imposes a mechanical barrier; the right-ward root skewing in *fer* might thus be a consequence of impaired mechano-sensing. Moreover, *fer* mutants are cellulose deficient [66] and this cell wall deficiency could potentially underlie the mechano-sensing defect in *fer*. Here, loss of MIK2 seems to lead to small, local cell wall defects as well as root skewing (Figs 3 and S7), suggesting that MIK2 could also be involved in mechano-sensing. Interestingly though, *fer* mutant roots skew right-ward, while *mik2* mutant roots do so left-ward, suggesting that different cell wall defects may translate into different root growth angles. Root skewing has also been previously reported in microtubules mutants [67–69]; however, we could not detect any difference in the orientation of cellulose microfibrils (S7A Fig), which align with the underlying cortical microtubules [59, 70–73], indicating that the root skewing phenotype observed in *mik2* plants is more complex. Future work should therefore address the molecular mechanisms underlying the observed root skewing.

Additionally, we found that *mik2* shows increased salt sensitivity (Fig 4). Mutants with altered cell wall composition or structure were previously shown to display enhanced NaCl sensitivity [74, 75]; the increased salt sensitivity of *mik2* mutants might thus be connected to its cell wall defects. In addition, we observed that *mik2* mutants display increased susceptibility to the hemi-biotrophic root pathogen *F*. *oxysporum* (Fig 5), while not to Arabidopsis leaf pathogens, such as the hemi-biotrophic bacterium *Pseudomonas syringae* pv. *tomato* DC3000, the obligate biotrophic oomycete *Hyaloperonospora arabidopsidis* Noco-2, or the necrotrophic fungus *Plectosphaerella cucumerina* BMM (S11A–S11C Fig). In addition, it was previously found that *mik2* mutant plants are not affected in resistance against the powdery mildew species *Golovinomyces orontii* and *Erysiphe pisi* [76]. We speculate that the role of MIK2 in *F*. *oxysporum* resistance is linked to a specific function in the root, which is possibly connected to CWI sensing.

Altogether, our results indicate that MIK2 is involved in a diverse array of biological processes in different tissues, similar to the candidate CWI sensor CrRLK1L FER that plays a role in cell elongation, mechano-sensing, pollen tube reception and immunity [8]. In all these processes, feedback information from the cell wall could play a potential important role. It is thus tempting to speculate that these diverse phenotypes of *mik2* and *fer* mutants are linked to a role in cell wall integrity sensing.

Up to now, one of the strongest candidate CWI sensors is the CrRLK1L THE1, as it is so far the only RK that displays impaired responses to cellulose biosynthesis inhibition [23]. FER and other malectin-like CrRLK1L family members have been proposed to play a role in CWI sensing based on the putative carbohydrate-binding capacity of their malectin domains, their structural resemblance to THE1, and their role in regulation of cell growth in diverse contexts [4, 6, 8]. In this study, we compared the phenotype of *mik2-1* with that of *the1-1*, and found that both RKs are required for responses to cellulose biosynthesis inhibition. However, differences exist in the extent to which these RKs regulate activation of immune marker genes and defence hormone production (Fig 2), suggesting these RKs might fulfil different functions. However, the function of MIK2 and THE1 seems to be linked, as the left-ward root skewing as well as enhanced salt sensitivity in *mik2-1* are abolished in *the1-1* genetic background (Figs 3 and 4). Intriguingly, *mik2-1* and *the1-1* seem to have distinct effects on cell wall structure in the root tip (S7 Fig), which could potentially underlie the observed root skewing and salt sensitivity in *mik2-1* and absence of thereof in *mik2-1 the1-1*. Loss of a cell wall sensor disrupts a cell wall-to-cell feedback loop; if such feedback information is lost, one could envision compensatory changes in cell wall composition and properties. Changes in non-cellulosic components can change the physical properties of the cell wall, and might thus affect the interaction between the root surface and the agar (*e*.*g*. the extent to which the root can resist the physical pressure of the agar could be different). This could subsequently influence the skewing angle under which the root grows, as well as its responses to external factors. We therefore hypothesize that loss of MIK2 results in mis-regulation of CWI sensing leading to local changes in cell wall composition that impact on root skewing and salt sensitivity. It is tempting to speculate that THE1 is required for these processes through sensing of a (cell wall-derived) signal in *mik2*. Alternatively, the lack of root skewing and salt sensitivity phenotypes in *mik2-1 the1-1* might result from changes in cell wall composition caused by loss of *THE1* that overrule changes caused by loss of *MIK2*. Of note is that cell wall disruption by inhibition of the cellulose synthase complex interfered with the root skewing response in *mik2-1* (Fig 3), which strengthens the hypothesis that root skewing is connected to cell wall changes. On the other hand, the observed effects of *mik2-1* and *the1-1* mutations on root growth direction, salt sensitivity, and cell wall structure could be consequences of another, potentially common, underlying cause. To distinguish between the different possibilities, additional insight into the type of cell wall changes that seem to occur in *mik2* versus *the1* mutant plants could prove useful. However, biochemical analysis of cell walls from whole roots did not reveal any significant changes in cell wall composition in the mutants compared to Col-0 (S9 Fig). The observed cell wall defects in *mik2-1* and *the1-1* might thus be subtler, local changes in the root tip, and will therefore be more difficult to detect in biochemical analysis.

Previously, LRR-RLKs FEI1 and FEI2 have been associated with CWI sensing [45]. However, opposite to *mik2*, the *fei1 fei2* double mutant shows increased sensitivity to inhibition of cellulose biosynthesis. Moreover, *fei1 fei2* is hypersensitive to high sucrose and high salt, and is disrupted in anisotropic cell expansion as well as in the synthesis of cell wall polymers [45]. These findings strengthen the suggestion that responsiveness to cellulose biosynthesis, cell wall composition and salt sensitivity are connected, and form another example of the involvement an LRR-RK in CWI sensing. However, the opposite effects of cellulose biosynthesis inhibition on *mik2* mutants compared with *fei1 fei2* suggest distinct roles for these proteins in CWI sensing.

Interestingly, we found that MIK2 is required for resistance against the root pathogen *F*. *oxysporum*, yet this role of MIK2 does not require THE1 (Fig 5). The effect of THE1 on *F*. *oxysporum* resistance seems therefore distinct from its effect on root growth direction and salt sensitivity. The exact role of THE1 in resistance to *F*. *oxysporum* remains to be determined, as we found discrepant results with two different alleles (Fig 5, S6D and S6E Fig). Of note is that *the1-4* has recently been suggested to be a gain-of-function, rather than a loss-of-function allele, which might explain the observed discrepancy [77]. Additional alleles would thus need to be tested. If THE1 is involved in resistance against *F*. *oxysporum*, MIK2 and THE1 might play a role through separate mechanisms. However, loss-of-function of both *MIK2* and *THE1* did not have an additive effect (Fig 5), suggesting that the two RKs could function in the same pathway. The putative role of THE1 in *F*. *oxysporum* resistance is clearly distinct from the related CrRLK1L FER, as an Arabidopsis mutant defective in FER has recently been shown to display enhanced resistance to *F*. *oxysporum*, most likely because FER is required for the perception of the secreted fungal RALF peptide that contributes to *F*. *oxysporum* virulence [39].

Excitingly, MIK2 was recently identified as part of the receptor complex for the female gametophyte-secreted peptide AtLURE1 that functions as a pollen tube attractant [46]. Moreover, *mik2* mutant plants displayed defects in male reproductive transmission and pollen tube guidance [46]. *AtLUREs* are part of a 6 gene-large species-specific cluster of defensin-like genes in Arabidopsis, expressed in the female gametophyte [78]. The Arabidopsis defensin-like gene family comprises 317 members [79]. Although other members of the AtLURE-receptor complex, MIK1, MALE DISCOVERER (MDIS) 1 and MDIS2, were not found to be involved in responses to ISX or in root skewing (S12 Fig), AtLUREs or related defensin-like peptides might be interesting ligand candidates for MIK2 during CWI, yet their role in CWI remains to be determined. It will be interesting to assess whether such peptides can be secreted/produced in response to cellulose biosynthesis inhibition, activate cellulose biosynthesis inhibition responses, and/or play a role in the control of root growth direction, salt tolerance and *F*. *oxysporum* resistance in an MIK2-dependent manner.

## Materials and methods

### Plant material

All *Arabidopsis thaliana* lines used in this study were in the Col-0 ecotype genetic background. The following mutants and transgenic lines were used: *ixr1-1* [50], *mik2-1* (SALK_061769), *mik2-2* (SALK_046987), *mik2-like-1* (SALK_112341C), *mik2-like-2* (GK-031G02-014862), *mik2-1 mik2-like-1*, *mik2-1 mik2-like-2*, *the1-1* (outcrossed from *prc1-1 the1-1* [23]), *the1-4* [25], *mik2-1 the1-1*, *GFP-CESA3 cesa3*^*je5*^ [82], *GFP-CESA3 cesa3*^*je5*^ *mik2-1*, *GFP-CESA3 cesa3*^*je5*^ *the1-1*, *prc1-1* [83], *mik2-1 prc1-1*, *the1-1 prc1-1*, *mik1* [46], *mdis1-2* [46], *mdis2* [46], and *mdis1-2 mdis2* [46].

### Genotyping

The following primers were used for genotyping of *mik2-1*, *mik2-2* and *mik2-like-1*:

*mik2-1* (SALK_061769) LP: 5’-AACGGATCGATTCCTTCTGA-3’
*mik2-1* (SALK_061769) RP 5’-TTTTGCCTGATAGCCGATTC-3’
*mik2-2* (SALK_046987) LP: 5’-GGAATCAGACTCTTAACCAA-3’
*mik2-2* (SALK_046987) RP: 5’-ACCCGACCCGACCATAACCG-3’
*mik2-like-1* (SALK_112341C) LP: 5’-CCACTCACTGGTATCATCCAAAACA-3’
*mik2-like-1* (SALK_112341C) RP: 5’-TCCGGTTAAGTGATTTGTGGA-3’
LBb1.3: 5’-ATTTTGCCGATTTCGGAAC-3’

Genotyping of *the1-1*, *prc1-1*, and *cesa3*^*je5*^ was performed by PCR amplification with the following primers:

*THE1* LP: 5’-AGCTTTTGGGTTTTCTTCGTTTTCC-3’
*THE1* RP: 5’-CTGTTTTGGAAAGTTATGTTTTGTGAGGAT -3’
*the1-1* LP: 5’-AGCTTTTGGGTTTTCTTCGTTTTCC-3’ (Same as *THE1* LP)
*the1-1* RP: 5’-CTGTTTTGGAAAGTTATGTTTTGTGACTAG-3’
*PRC1* LP: 5’-ATCGAAGAGGGCCGCGTCA-3’
*PRC1* RP: 5’-ACTGCCCAAATTTCTTCTCCAACTTCAATT-3’
*cesa3*^*je5*^ LP: 5’-CAGGTTTGACACCTCTCTCT-3’
*cesa3*^*je5*^ RP: 5’-GTCCGGTTCTGTCGACCCAT-3’

Next, PCR products were digested with BamHI (Invitrogen, Carlsbad, CA, USA) (cuts *THE1*), SpeI (Roche, Basel, Switzerland) (cuts *the1-1*), MfeIHF (New England Biolabs, Ipswich, MA, USA) (cuts *PRC1*), and HphI (New England Biolabs) (cuts *cesa3*^*je5*^) for 4 h at 37°C following manufacturer’s instructions. Digested PCR products were separated on a 3% agarose gel in TBE (for *THE1*/*the1-1* and *PRC1/prc1-1*) or 1% agarose in TBE (for *CESA3*/*cesa3*^*je5*^).

### Cloning

The MIK2 coding sequence was amplified from Col-0 cDNA using the primers 5’-CACCATGAACAAAACAAACCCAG-3’ and 5’-AGAAAAGGCAGTGGAGATAGAGAGC-3’. The corresponding amplicon was cloned into pENTR/D-TOPO using the pENTR Directional TOPO Cloning Kit (Invitrogen, CA, USA). The insert was then transferred into the Gateway-compatible binary vector pEarleyGate103 [84] using GATEWAY LR CLONASE II enzyme (Invitrogen). The final construct was electroporated into *Agrobacterium tumefaciens* strain GV3101 [85].

### RNA extraction and qPCR analysis

For gene expression analysis, seeds were sown on full strength Murashige and Skoog (MS) medium (4.41 g/L; including vitamins; Duchefa, Haarlem, The Netherlands) and 1% sucrose supplemented with 0.8% agar. The seeds were stratified for 2 days at 4°C, and incubated for 5 days at 22°C under a 16-h photoperiod. Seedlings were then transferred to liquid MS medium with 1% sucrose, and grown for another 7 days, after which the growth medium was refreshed. Next day, plants were mock treated, or treated with 0.6 μM isoxaben (ISX) (Sigma-Aldrich, St. Louis, MO, USA), 6 μM 2,6-dichlorobenzonitrile (DCB) (Sigma-Aldrich), 0.4 μM thaxtomin (TXT) (Sigma-Aldrich), or 400 mM mannitol as indicated in the figures. ISX and DCB were added from respectively 1.2 mM and 12 mM stocks in DMSO; TXT was added from a 800 μm stock in 100% ethanol. All treatments contained equal amounts of DMSO and ethanol. Total RNA was extracted using Trizol reagent (Invitrogen) according to the manufacturer’s instructions. RNA samples were treated with Turbo DNA-free DNase (Ambion/Thermo fisher Scientific, Waltham, MA, USA) according to the manufacturer’s instructions. RNA was quantified with a Nanodrop spectrophotometer (Thermo fisher Scientific). cDNA was synthesized from 5 μg RNA using SuperScript III Reverse Transcriptase (Invitrogen/Thermo fisher Scientific) according to the manufacturer’s instructions. cDNA was amplified by quantitative PCR using SYBR Green JumpStart Taq ReadyMix (Sigma-Aldrich) and the PTC-200 Peltier Thermal Cycler (Bio-Rad Laboratories, Hercules, CA, USA). The relative expression values were determined using *U-box* as reference and the comparative Ct method (2-ΔΔCt). The following primers were used for quantitative RT-PCR:

*U-box (At5g15400*) LP: 5′-TGCGCTGCCAGATAATACACTATT-3′ [86]
*U-box* (*At5g15400*) RP: 5′-TGCTGCCCAACATCAGGTT-3′ [86]
*MIK2*.*1* (*At4g08850*.*1*) LP: 5’-CTATGTTGCTCCAGAACTAG-3’
*MIK2*.*1* (*At4g08850*.*1*) RP: 5’-GTTCCGGTAGCCGGTGGTCG-3’
*MIK2*.*2* (*At4g08850*.*2*) LP: 5’-CTATGTTGCTCCAGgtacg-3’
*MIK2*.*2* (*At4g08850*.*2*) RP: 5’-ACCCGACCCGACCATAACCG-3’
*MIK2-LIKE* (*At1g35710*) LP: 5’-CAACGTTTCGAAAAGCAACA-3’
*MIK2-LIKE* (*At1g35710*) RP: 5’-TGCCATTTTTCTTCGGTTTC-3’
*FRK1* (*At2g19190*) LP: 5′-ATCTTCGCTTGGAGCTTCTC-3′ [49]
*FRK1* (*At2g19190*) RP: 5′-TGCAGCGCAAGGACTAGAG-3′ [49]
*At1g51890* LP: 5′-CCAGTTTGTTCTGTAATACTCAGG-3′ [49]
*At1g51890* RP: 5′-CTAGCCGACTTTGGGCTATC-3′ [49]
*CYP81F2* (*At5g57220*) LP: 5′-AATGGAGAGAGCAACACAATG-3′ [49]
*CYP81F2* (*At5g57220*) RP: 5′-ATACTGAGCATGAGCCCTTTG-3′ [49]

### Quantification of JA, SA and lignin deposition

Arabidopsis seedlings were grown in liquid culture as described in [9]. Six day-old seedlings were brought into new flasks with growth medium supplemented with either DMSO (mock) or 0.6 μM ISX. At 7 h after treatment, seedlings were harvested in liquid N_2_ and JA and SA were extracted and measured as described [87]. At 12 h after treatment, seedlings were harvested in 70% EtOH and stained for lignification using phloroglucinol-HCl as described in [9]. For determination of lignin deposition in the root elongation zone, pictures were taken with a Zeiss Axio Zoom.V16 stereo microscope. Phlorogucinol-stained areas were quantified using ImageJ software and normalized to the total root area photographed, while the root length was kept equal in all images. The ratios obtained are plotted as fold change compared to Col-0.

### Hypocotyl growth elongation assays

Seeds were sown on square plates with full strength MS medium (4.41 g/L; including vitamins; Duchefa) and 1% sucrose supplemented with 0.8% agar. The seeds were stratified for 2 days at 4°C, and incubated for 5 days at 22°C in the dark, in an upright position.

### Root skewing assays

Seeds were sown on square plates with full strength MS medium (4.41 g/L; including vitamins; Duchefa) and 1% sucrose supplemented with 0.8% agar. Where indicated in the figures, growth medium contained DMSO (mock), 2 nM ISX (Sigma-Aldrich), or 25 μM DCB (Sigma-Aldrich). ISX and DCB were added from respectively 80 μM and 1 mM stocks in DMSO. All treatments contained equal amounts of DMSO. The seeds were stratified for 2 days at 4°C, and incubated for 9 days at 22°C under a 16-h photoperiod, in an upright position under a 10° angle relative to the direction of gravity.

### Biochemical analysis of the cell wall

Seeds were sown on full strength MS medium (4.41 g/L; including vitamins; Duchefa) and 1% sucrose supplemented with 0.8% agar. The seeds were stratified for 2 days at 4°C, and incubated for 5 days at 22°C under a 16-h photoperiod. Seedlings were then transferred to liquid MS medium with 1% sucrose, and grown for another 2 days, after which the plants were mock treated, or treated with 0.6 μM ISX (Sigma-Aldrich) for 5 h. ISX was added from a 1.2 mM stock in DMSO. Mock and ISX treatment contained an equal amount of DMSO. Seedlings were harvested in 100% ethanol. Root and shoot tissue was separated, 100 roots were used per sample. Root tissue was washed once in ethanol and twice in acetone, and roots were dried overnight.

Galacturonic acid content of a Homogalacturonan enriched fraction was determined by incubation of the roots with 100 μL 1% ammonium oxalate (pH 5) for 2 h at 80°C, shaking at 300 rpm. The supernatant was collected, samples were diluted 10 times, and sulfuric acid was added (1.5 mL sulfuric acid per 250 μL sample in glass tubes). Samples were incubated for 15 min at 100°C, kept on ice for 5 minutes. Galacturonic acid content was then measured following the method described in [88], adapted from [89]. A standard range of galacturonic acid (0–0.1 g/L) was included to calculate uronic acid concentration. Cellulose and monosaccharide levels were determined as described [90].

### Fourier-transform infrared (FT-IR) spectroscopy

Seedlings were grown and treated as described under “Biochemical analysis of the cell wall”. Seedlings were harvested in ethanol. One day prior to measuring, ethanol was replaced by milliQ water. Seedlings were mounted on gold coated glass slides (Thermo fisher Scientific) and dried for 20 min at 37°C. Per root, 20 adjacent areas of 40 μm by 40 μm along the lowest 800 μm of the root, on the side of the central cylinder were selected for spectra collection. Per sample 4 roots were measured, and the experiment was repeated 4 times. Spectra were collected and normalized as described [91]. Statistical analysis was performed using a Student’s *T*-test with “R” software as described [92].

### Imaging of MIK2-GFP in *N*. *benthamiana*

*A*. *tumefaciens* strains carrying MIK2-GFP (pEarleyGate103/*35S*::*MIK2-GFP-6xHis*) was used for transient expression in *N*. *benthamiana*. Transient expression and imaging was realized as described [93]. Cell plasmolysis was induced by treatment with 1 M NaCl for 20 min.

### Imaging of GFP-CESA3

Seeds were sown on square plates with full strength MS medium (4.41 g/L; without vitamins; Duchefa) and 1% sucrose supplemented with 0.8% agar. The seeds were stratified for 2 days at 4°C, and plates were incubated in an upright position for 4 days at 22°C under a 16-h photoperiod. Seedlings were transferred to liquid MS medium with 1% sucrose, and were mock treated, or treated with 0.1 μM ISX (Sigma-Aldrich) for 2 h. ISX was added from a 0.1 mM stock in DMSO. Mock and ISX treatment contained an equal amount of DMSO. GFP-CESA3 was imaged as described previously [94].

### Imaging of cellulose microfibrils

Seedlings were grown as described under “Imaging of GFP-CESA3”, yet here seedlings were grown for 7 days. Pontamine Fast Scarlet 4B staining was performed as described in [94], with some modifications. Seedlings were fixed under vacuum in 4% paraformaldehyde in 0.5 X MTSB buffer with 0.1% Triton for 1 h. Seedlings were washed in 1 X PBS, and incubated overnight at room temperature in 0.003% Pontamine Fast Scarlet 4B (Sigma-Aldrich) in 1 X PBS. Next, seedlings were washed with 1 X PBS, mounted in 20 μg/mL citifluor/DAPI, and imaged using the 514-nm laser line of a SP5 confocal laser scanning microscope (Leica, Solms, Germany) equipped with an argon laser, as described in [94]. The orientation of cellulose microfibrils relative to the direction of cell elongation was quantified using ImageJ software. Values from 3 independent experiments were combined; per genotype values of 10 roots were collected, and per root a minimum of 12 cells were measured.

### Imaging of root tip cells stained with propidium iodide

Imaging of root tip cells stained with propidium iodide was performed as described [95].

### Salt tolerance assays

The change in root angle in response to salt or sorbitol was determined in seedlings grown on agar plates under a 16-h photoperiod. Plants were germinated on ½ MS medium without sucrose. After 4 days, plants were transferred to new medium with 0 mM or 75 mM of NaCl, or 150 mM of sorbitol (comparable in osmolarity to 75 mM of NaCl). Six days after transfer (10-day-old seedlings), plates were scanned with an Epson scanner from below. Roots were traced with SmartRoot (plugin in ImageJ software) and the directionality output was used to determine the angle of the root (after transfer). The experiment was repeated three times with similar results.

For determination of salt tolerance, plants were grown in pots under an 11-h photoperiod, at 22 degrees and 70% humidity. One week after germination, plants were transferred to pots which were saturated with 4 L of either 0 or 75 mM of NaCl solution. During the experiment, all plants were watered with rainwater from below. Conductivity measurements confirmed that salt levels stayed stable during the experiment. After 4 weeks of treatment, plants were cut off and dried in an oven on 68 degrees for 1 week to determine dry weight. Plants were randomised over trays using a randomized block design. Randomisation was similar for each treatment. The experiment was repeated three times with similar results.

### Infection experiments

*F*. *oxysporum* (strain Fo5176; originally isolated by Queensland Plant Pathology Herbarium, Queensland Department of Primary Industries and Fisheries, Brisbane, Australia) was grown on Czopek-Dox-Agar medium. To obtain spores, an agar plug was added to liquid medium consisting of 3% sucrose, 100 mM KNO_3_ and 0,17% yeast nitrogen base and incubated on a shaker for 3 days. Spores were harvested by filtrating through miracloth, washed and diluted with water. 2-week-old Arabidopsis plants were inoculated by pipetting 750 μL spore solution (10^7^ spores/ml) 1–2 cm deep into the soil, directly next to a plant. Subsequently plants were grown in a climate chamber at 11-hour light/ 13-hour dark cycle, 28°C and 80% relative humidity. The number of chlorotic leaves was counted 12 days post inoculation, and the number of decayed plants estimated 3 weeks post inoculation.

*Pseudomonas syringae* pv. *tomato* DC3000 infections were carried out on 4-week-old plants. Overnight bacterial culture was pelleted and resuspended in 10 mM MgCl_2_ to an OD_600_ of 0.02 in presence of 0.02% (v/v) Silwet L-77. Bacteria were sprayed onto leaf surfaces, and plants were maintained covered. Two days post-inoculation, leaf discs were sampled and ground in 10 mM MgCl_2_. After dilution and plating on Luria-Bertani agar with appropriate selection, plates were incubated at 28°C and colonies were counted 2 days later.

*P*. *cucumerina* BMM inoculation was carried out on 18-day-old soil-grown plants by spraying a suspension of 4x10^6^ spores/mL of the fungus. Disease progression in the inoculated plants was estimated by an average disease symptom (0–5) as previously described [96].

Inoculations with spore suspensions of *Hyaloperonospora arabidopsidis* Noco2 isolate (5x10^4^ spores/mL) were performed on 11-day-old seedlings grown under short day conditions. Progression of the infection was scored after 7 days as previously described [97].

## Supporting information

S1 Fig Characterization of MIK2. (A) Gene models for *MIK2* indicating the positions of the T-DNA insertions (yellow triangles), and the primers (green arrows) used for detection of *MIK2*.*1* and *MIK2*.*2*. (B,C) *MIK2*.*1* and *MIK2*.*2* (B) and immune marker gene (C) expression in 13-day-old Arabidopsis seedlings determined by qRT-PCR. *MIK2*.*1* is the more abundant splice form; in whole seedlings it is 8–50 fold higher expressed than *MIK2*.*2*. (C) Seedlings were mock treated or treated with 0.6 μM ISX for 9 h. (B,C) Error bars represent standard error of three technical replicas. The experiments were repeated three times with similar results. Asterisks indicate a statistically significant difference relative to Col-0, as determined by a two-tailed Student’s *T*-test (*p* < 0.05). (D) Protein model for MIK2.1. (E) Confocal images of MIK2.1-GFP in *N*.*benthamiana*. MIK2.1-GFP localizes to the plasma membrane before (left panel) and after plasmolysis induced by treatment with 1 M NaCl for 20 min (right panel). (F) Nine-day-old Arabidopsis seedlings grown in an upright position (under a 10° angle relative to the direction of gravity) on MS agar medium with 1% sucrose. Root angle relative to the vertical growth axis, and root length were quantified. Error bars represent standard error of n = 15 biological replicas. The experiment was repeated three times with similar results. Different letters indicate statistically significant differences between genotypes (ANOVA and Holm-Sidak test (*p* < 0.05)).
(TIF)

S2 Fig The role of MIK2-LIKE in responses triggered by cellulose biosynthesis inhibition and control of root growth angle. (A) Phylogenetic tree based on homology in the C-terminal domain of MIK2 proteins in *Arabidopsis thaliana (A*.*t*.*)*, *Arabidopsis lyrata (A*.*l*.*)* and *Brassica rapa (B*.*r*.*)*. Regions homologous to *Arabidopsis thaliana* MIK2 amino acids 620–1045 were aligned, and a tree was drawn using CLC Main Workbench 7.0.3 software. (B) Gene model for *MIK2-LIKE* indicating the position of the T-DNA insertions (yellow triangles), and the primers (green arrows) used for detection of *MIK2-LIKE*. (C,D) *MIK2-LIKE* (C) and immune marker gene (D) expression in 13-day-old Arabidopsis seedlings determined by qRT-PCR. (D) Seedlings were mock treated, or treated with 0.6 μM ISX for 9 h. Expression of the immune marker gene *CYP81F2* was normalized relative to *U-box* expression values. Depicted is the fold change in expression relative to mock treatment. (C,D) Error bars represent standard error of three technical replicas. (E,F) JA production (E) and lignin-deposition (F) in 6-day-old Arabidopsis seedlings, mock treated or treated with 0.6 μM ISX for 7 h (E) and 12 h (F). Error bars represent standard error of n = 4 biological replicas. (E) The upper and lower panel display the same data, yet in the lower panel, the y-axis has been adjusted to better visualize the JA levels in mock-treated samples. (F) The average of 4 independent experiments is shown. In each experiment lignification values in Col-0 were set at 1. (C-F) Asterisks indicate a statistically significant difference relative to Col-0 (*p* < 0.05 (C,D,F)), or a near significant difference *p* = 0.06 (E)), as determined by a two-tailed Student’s *T*-test (G) Nine-day-old Arabidopsis seedlings grown in an upright position (under a 10° angle relative to the direction of gravity) on MS agar medium with 1% sucrose. Root angle relative to the vertical growth axis was quantified. Error bars represent standard error of n = 15 biological replicas. Different letters indicate statistically significant differences between genotypes (ANOVA and Tukey HSD test (*p* < 0.05)). (C-G) The experiments were repeated at least three times with similar results.
(TIF)

S3 Fig *MIK2*, *MIK2-LIKE* and *THE1* expression in different organs. Expression of *MIK2*, *MIK2-LIKE*, and *THE1* in different organs [80].
(TIF)

S4 Fig MIK2 is not required for hypocotyl growth reduction in *prc1-1* genetic background. Five-day-old seedlings grown in an upright position in the dark on MS agar medium supplemented with 1% sucrose. Hypocotyl length was quantified. Error bars represent standard error of n = 18 biological replicas. Different letters indicate statistically significant differences between genotypes (ANOVA and Tukey HSD test (*p* <0.05)). The experiment was repeated six times with similar results.
(TIF)

S5 Fig ISX-induced CESA3 internalization in *mik2-1* and *the1-1* mutant background. (A,B) Confocal images of GFP-CESA3 in *cesa3*^*je5*^, *cesa3*^*je5*^ *mik2-1*, or *cesa3*^*je5*^ *the1-1* genetic background. Four-day-old Arabidopsis seedlings were mock treated or treated with 0.1 μM ISX for 2 h. Panel A displays the cell surface, while panel B displays a cross section through the cells. ISX treatment results in internalization of GFP-CESA3; GFP-CESA3 accumulates in microtubule-associated cellulose synthase compartments (MASCs) in the cell cortex. In panel A the red arrows indicate GFP-CESA3 in MASCs. In panel B the yellow arrows indicate the position of the plasma membrane, which is rich in GFP-CESA3 signal upon mock treatment and depleted of GFP-CESA3 after ISX treatment. The large circular fluorescent organelles are GFP-CESA3 signal in the Golgi apparatus. The size bars represent 10 μm. (C) Quantification of the surface particles depicted in (A). Asterisks indicate a statistically significant difference as determined by a two-tailed Student’s *T*-test (*p* < 0.05). Error bars represent the standard error of n = 80 measurements in 15 seedlings. The particle density analysis was performed as described [81].
(TIF)

S6 Fig The role of THE1 in control of root growth angle, salt tolerance and resistance to *F*. *oxysporum*. (A) Nine-day-old Arabidopsis seedlings grown in an upright position (under a 10° angle relative to the direction of gravity) on MS agar medium with 1% sucrose. Root angle relative to the vertical growth axis, and root length were quantified. Error bars represent standard error of n = 15 biological replicas. (B) Ten-day-old Arabidopsis seedlings were grown in an upright position on ½ MS agar medium without sucrose, supplemented with or without 75 mM NaCl or 150 mM sorbitol. Depicted is the change in the angle of the root after NaCl or sorbitol treatment compared to mock treatment; the negative value indicates a change to the right. Error bars represent standard error of n = 20 biological replicas. (C) Dry weight of NaCl-treated plants as percentage of the dry weight of untreated plants. Plants were treated as described in Fig 4. Error bars represent the standard error of n = 20 plants. An asterisk indicates a significant difference from Col-0 according to a linear mixed model (*p* < 0.05) (D,E) Percentage of chlorotic leaves per plant (D), and percentage of decayed plants (E) after infection of the roots with *F*. *oxysporum* isolate Fo5176. The experiment was performed as described in Fig 5. The bars represent the average of three independent experiments, each consisting of n = 20–40 plants per genotype. Error bars represent the standard error of n = 3 experiments. No disease symptoms were observed on mock-inoculated plants for any of the genotypes (n = 10). (A,B,D,E) Different letters indicate statistically significant differences between genotypes (ANOVA and Tukey HSD test (*p* < 0.05)). The experiments were repeated at least three times with similar results.
(TIF)

S7 Fig *Mik2* and *the1* have distinct effects on cell wall structure in the root tip. (A) Quantification of the orientation of cellulose microfibrils relative to the direction of cell elongation in root tips of 7-day-old Arabidopsis seedlings. Values of 3 independent experiments were combined. Error bars represent standard error of n = 10 roots. (B,C) FT-IR spectroscopy of root tips of 7 days-old Arabidopsis seedlings. Absorption spectra were collected along 800 μm of the root tip, spanning the elongation zone and the beginning of the differentiation zone. Absorption spectra of 4 independent experiments were combined and spectra of *mik2-1*, *the1-1*, and *mik2-1 the1-1* were compared with Col-0. (B) *T*-test values for the indicated comparisons. *T*-test values above 2 or below -2 (marked by red lines) indicate statistically significant differences (*p* < 0.01). (C) Average absorbance spectra. Wavenumbers of the main 4 peaks are indicated in black. (B,C) Asterisks high-light points were mutants differ significantly from Col-0; corresponding wavenumbers are indicated in red.
(TIF)

S8 Fig Root tip morphology in *mik2-1*, *the-1* and *mik2-1 the1-1*. *mik2-1*, *the1-1* and *mik2-1 the1-1* mutants do not display any apparent defects in phloem continuity or root meristem morphology. (A) Confocal microscopy pictures of the root meristem of 7-day-old seedlings of the indicated genotypes stained with propidium iodide (red). Protophloem is visible as a bright, uninterrupted strand within the stele. (B) Cross sections of the root meristem of 5-day-old seedlings of the indicated genotypes, stained with toluidine blue. The number of cell files in the stele is quantified in (C) (n≥14; the mutant values are not significantly different from the Col-0 control [student’s t-test]).
(TIF)

S9 Fig Biochemical analysis of cell wall composition in Col-0, *mik2-1*, *the1-1*, and *mik2-1 the1-1* plants. Levels of cellulose, pectin (galacturonic acid (GA)), and monosaccharides derived from hemi-cellulose or pectin, in roots of 7-day-old Arabidopsis seedlings. Values are expressed per mg root tissue. Depicted is the average of four independent experiments, and error bars represent standard error. Different letters indicate a statistically significant difference between genotypes (ANOVA followed by Tukey HSD test (*p* < 0.05)).
(TIF)

S10 Fig Dry weight of *mik2-1*, *the1-1* and *mik2-1 the1-1* after mock or NaCl treatment. Dry weight of Arabidopsis plants treated with or without NaCl, as described in Fig 4B. Different letters indicate statistically significant differences between genotypes (Left panel: ANOVA and Holm-Sidak test (*p* < 0.05), right panel: Kruskal-Wallis ANOVA on ranks followed by Dunn’s multiple comparison procedures (*p* <0.05)).
(TIF)

S11 Fig Assessment of susceptibility of the *mik2-1* mutant to bacterial and fungal pathogens. (A) Growth of *Pseudomonas syringae* pv. *tomato* DC3000 in Col-0 and *mik2-1* mutant plants. The hypersusceptible mutant *fls2c* was included as a control. Plants were sprayed with a *P*. *syringae* bacterial suspension (OD_600_ = 0.02), and material was harvested two days later for quantification of bacterial growth. (B) Plant disease rating at different days post inoculation (dpi) with the necrotrophic fungus *Plectosphaerella cucumerina* BMM *(Pc*BMM*)*. Three-week-old Arabidopsis Col-0 plants, the *mik2-1* mutant, and the *irx1-6* and *agb1-1* mutants, included as resistant and hypersusceptible controls, respectively, were inoculated with 4 x 10^6^ spores/mL of *PcBMM*. Quantification of fungal growth was estimated by visual evaluation of the plant disease symptoms (from 0 to 5) and average disease rating was determined. Values are means ± standard deviation (n = 10). (C) Resistance to the biotrophic pathogen *Hyaloperonospora arabidospsidis (Hpa)*. Two-week-old plants of the indicated genotypes and the *Hpa* hypersusceptible *eds1-2* mutant were inoculated with 5 x 10^4^ spores/mL *Hpa*. Fungal growth in leaves was determined 7 dpi by measuring *Hpa* sporulation (*Hpa* spores/mg plant fresh weight (fw)). Values are means ± standard deviation (n = 10). (A-C) The experiments were repeated at least three times with similar results. Statistically significant values (*) that differ from those of wild-type plants were determined by Student’s *T-*test *(p* < 0.05).
(TIF)

S12 Fig The role of atlure receptor complex components in response to cellulose biosynthesis inhibition and control of root growth angle. (A) Immune marker gene expression in 13-day-old Arabidopsis seedlings determined by qRT-PCR. Seedlings were mock treated, or treated with 0.6 μM ISX for 9 h. Expression of the immune marker gene *CYP81F2* was normalized relative to *U-box* expression values. Depicted is the fold change in expression relative to mock treatment. Error bars represent standard error of three technical replicas. The asterisk indicates a statistically significant difference relative to Col-0, as determined by a two-tailed Student’s *T*-test (*p* < 0.05). (B) Nine-day-old Arabidopsis seedlings grown in an upright position (under a 10° angle relative to the direction of gravity) on MS agar medium with 1% sucrose. Root angle relative to the vertical growth axis was quantified. Error bars represent standard error of n = 15 biological replicas. Different letters indicate statistically significant differences between genotypes (ANOVA and Tukey HSD test (*p* < 0.05)). (A,B) The experiments were repeated at least three times with similar results.
(TIF)
