# Supplementary figures and images for: The Arabidopsis leucine-rich repeat receptor kinase MIK2/LRR-KISS connects cell wall integrity sensing, root growth and response to abiotic and biotic stresses
Source: PLoS Genet. 2017 Jun 12;13(6):e1006832. doi: 10.1371/journal.pgen.1006832 (PMC5484538; doi:10.1371/journal.pgen.1006832)

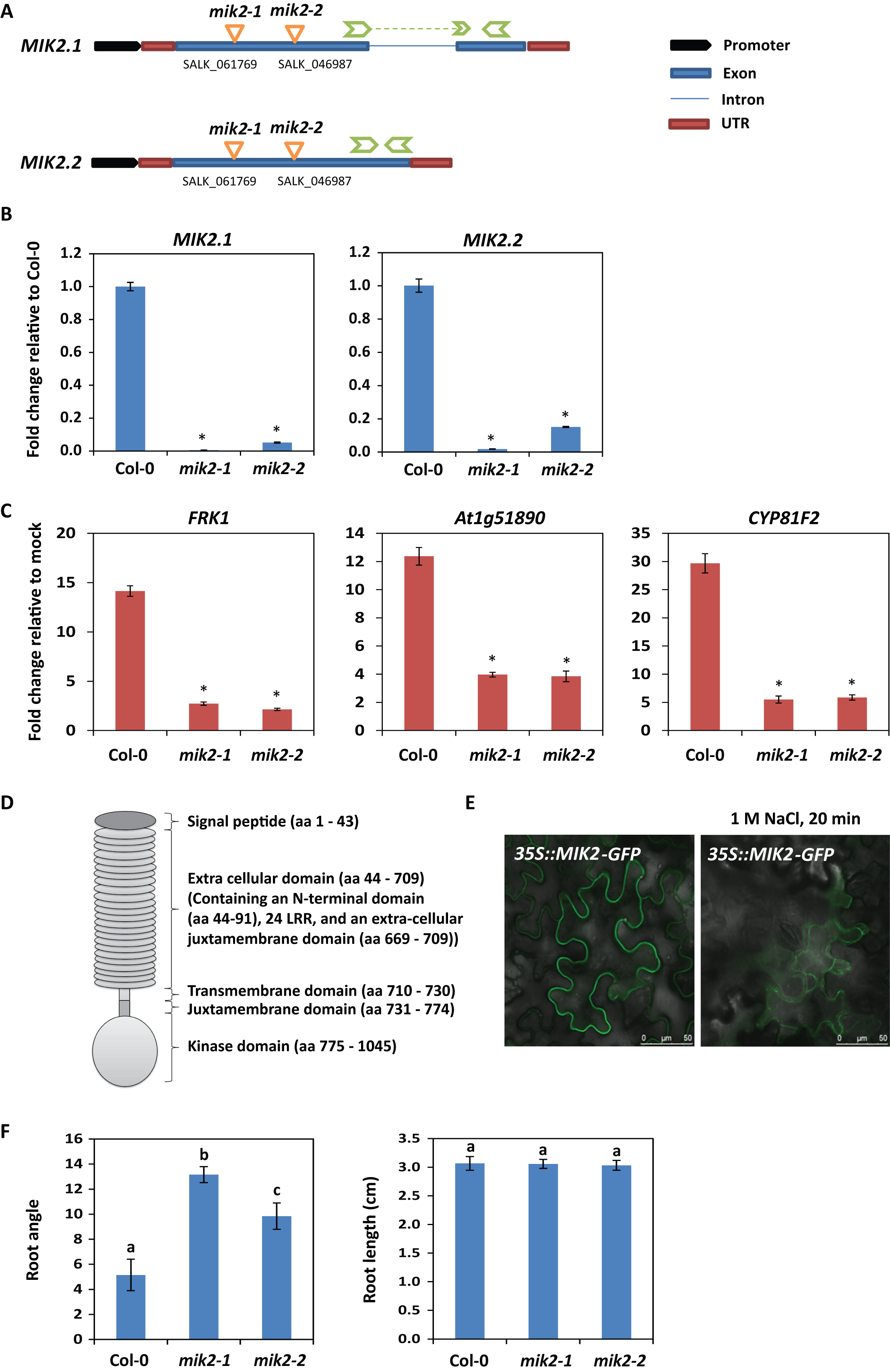

Supplement: S1 Fig — (A) Gene models for MIK2 indicating the positions of the T-DNA insertions (yellow triangles), and the primers (green arrows) used for detection of MIK2.1 and MIK2.2. (B,C) MIK2.1 and MIK2.2 (B) and immune marker gene (C) expression in 13-day-old Arabidopsis seedlings determined by qRT-PCR. MIK2.1 is the more abundant splice form; in whole seedlings it is 8–50 fold higher expressed than MIK2.2. (C) Seedlings were mock treated or treated with 0.6 μM ISX for 9 h. (B,C) Error bars represent standard error of three technical replicas. The experiments were repeated three times with similar results. Asterisks indicate a statistically significant difference relative to Col-0, as determined by a two-tailed Student’s T-test (p < 0.05). (D) Protein model for MIK2.1. (E) Confocal images of MIK2.1-GFP in N.benthamiana. MIK2.1-GFP localizes to the plasma membrane before (left panel) and after plasmolysis induced by treatment with 1 M NaCl for 20 min (right panel). (F) Nine-day-old Arabidopsis seedlings grown in an upright position (under a 10° angle relative to the direction of gravity) on MS agar medium with 1% sucrose. Root angle relative to the vertical growth axis, and root length were quantified. Error bars represent standard error of n = 15 biological replicas. The experiment was repeated three times with similar results. Different letters indicate statistically significant differences between genotypes (ANOVA and Holm-Sidak test (p < 0.05)). (TIF) [file pgen.1006832.s001.tif]

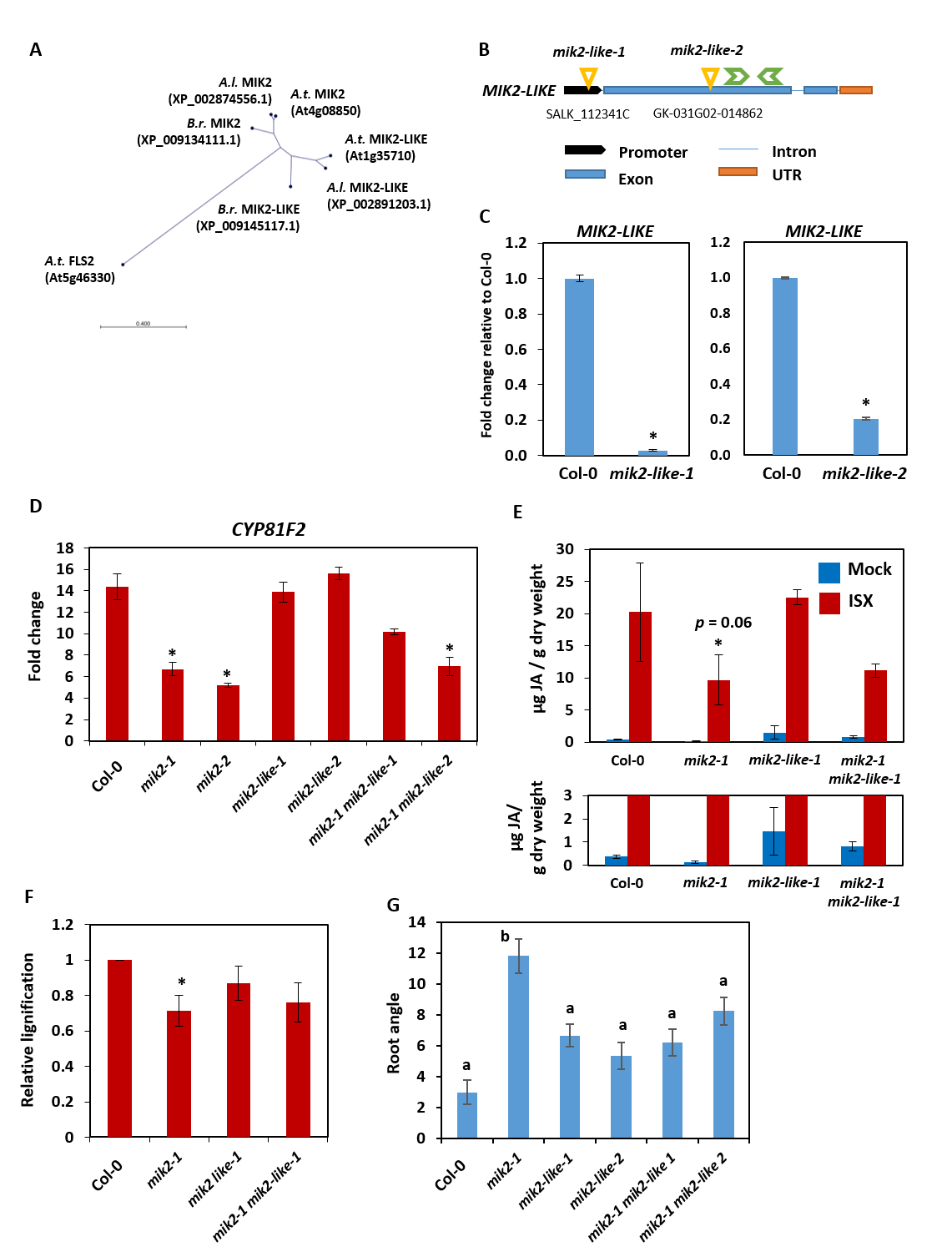

Supplement: S2 Fig — (A) Phylogenetic tree based on homology in the C-terminal domain of MIK2 proteins in Arabidopsis thaliana (A.t.), Arabidopsis lyrata (A.l.) and Brassica rapa (B.r.). Regions homologous to Arabidopsis thaliana MIK2 amino acids 620–1045 were aligned, and a tree was drawn using CLC Main Workbench 7.0.3 software. (B) Gene model for MIK2-LIKE indicating the position of the T-DNA insertions (yellow triangles), and the primers (green arrows) used for detection of MIK2-LIKE. (C,D) MIK2-LIKE (C) and immune marker gene (D) expression in 13-day-old Arabidopsis seedlings determined by qRT-PCR. (D) Seedlings were mock treated, or treated with 0.6 μM ISX for 9 h. Expression of the immune marker gene CYP81F2 was normalized relative to U-box expression values. Depicted is the fold change in expression relative to mock treatment. (C,D) Error bars represent standard error of three technical replicas. (E,F) JA production (E) and lignin-deposition (F) in 6-day-old Arabidopsis seedlings, mock treated or treated with 0.6 μM ISX for 7 h (E) and 12 h (F). Error bars represent standard error of n = 4 biological replicas. (E) The upper and lower panel display the same data, yet in the lower panel, the y-axis has been adjusted to better visualize the JA levels in mock-treated samples. (F) The average of 4 independent experiments is shown. In each experiment lignification values in Col-0 were set at 1. (C-F) Asterisks indicate a statistically significant difference relative to Col-0 (p < 0.05 (C,D,F)), or a near significant difference p = 0.06 (E)), as determined by a two-tailed Student’s T-test (G) Nine-day-old Arabidopsis seedlings grown in an upright position (under a 10° angle relative to the direction of gravity) on MS agar medium with 1% sucrose. Root angle relative to the vertical growth axis was quantified. Error bars represent standard error of n = 15 biological replicas. Different letters indicate statistically significant differences between genotypes (ANOVA and Tukey HSD test (p < [file pgen.1006832.s002.tif]

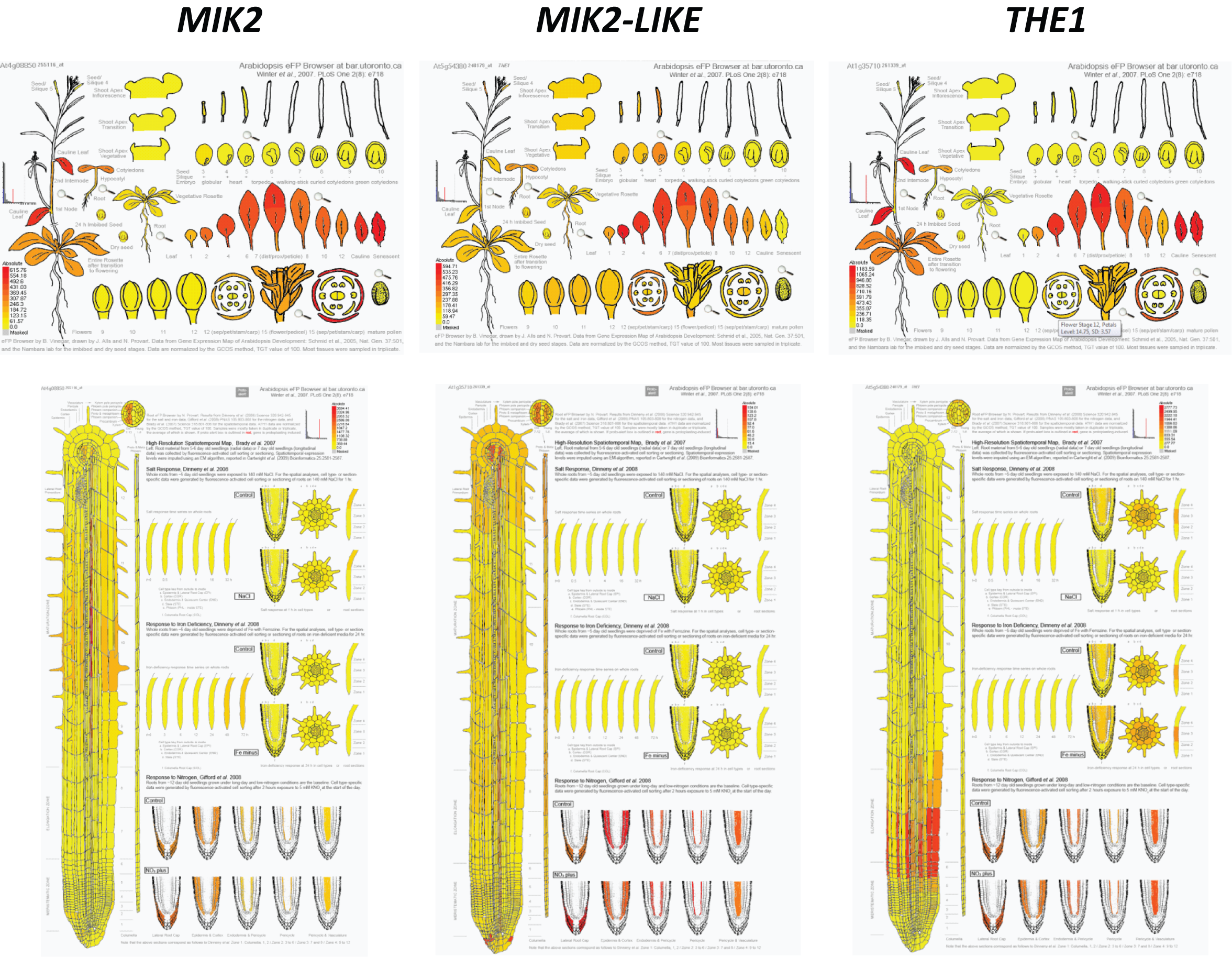

Supplement: S3 Fig — Expression of MIK2, MIK2-LIKE, and THE1 in different organs [80]. (TIF) [file pgen.1006832.s003.tif]

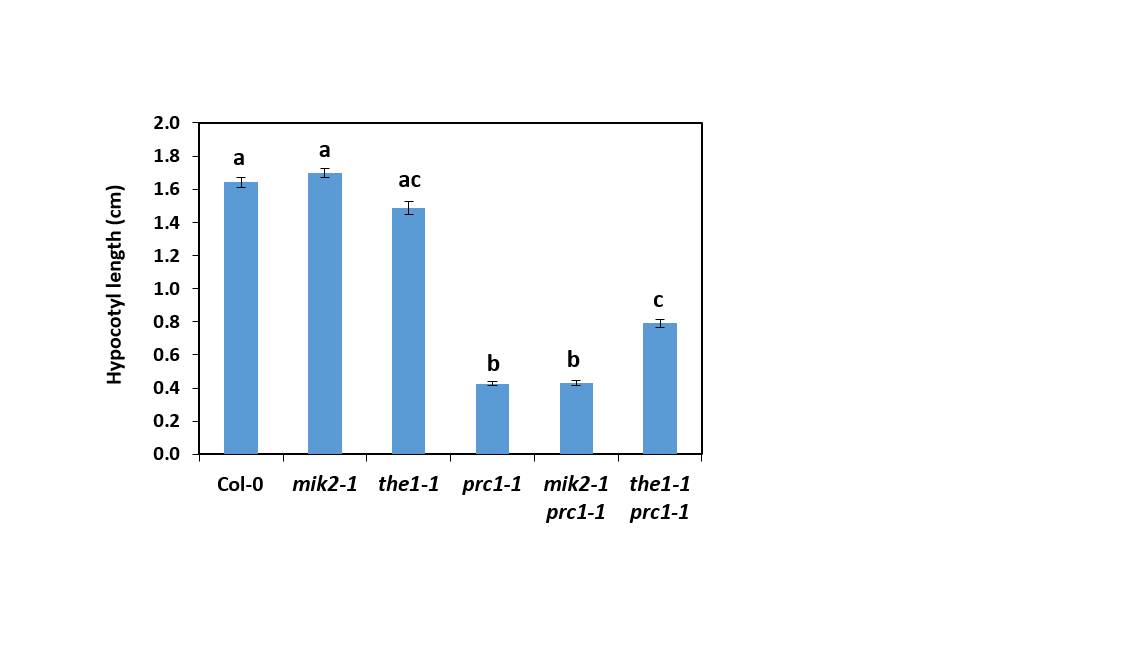

Supplement: S4 Fig — Five-day-old seedlings grown in an upright position in the dark on MS agar medium supplemented with 1% sucrose. Hypocotyl length was quantified. Error bars represent standard error of n = 18 biological replicas. Different letters indicate statistically significant differences between genotypes (ANOVA and Tukey HSD test (p <0.05)). The experiment was repeated six times with similar results. (TIF) [file pgen.1006832.s004.tif]

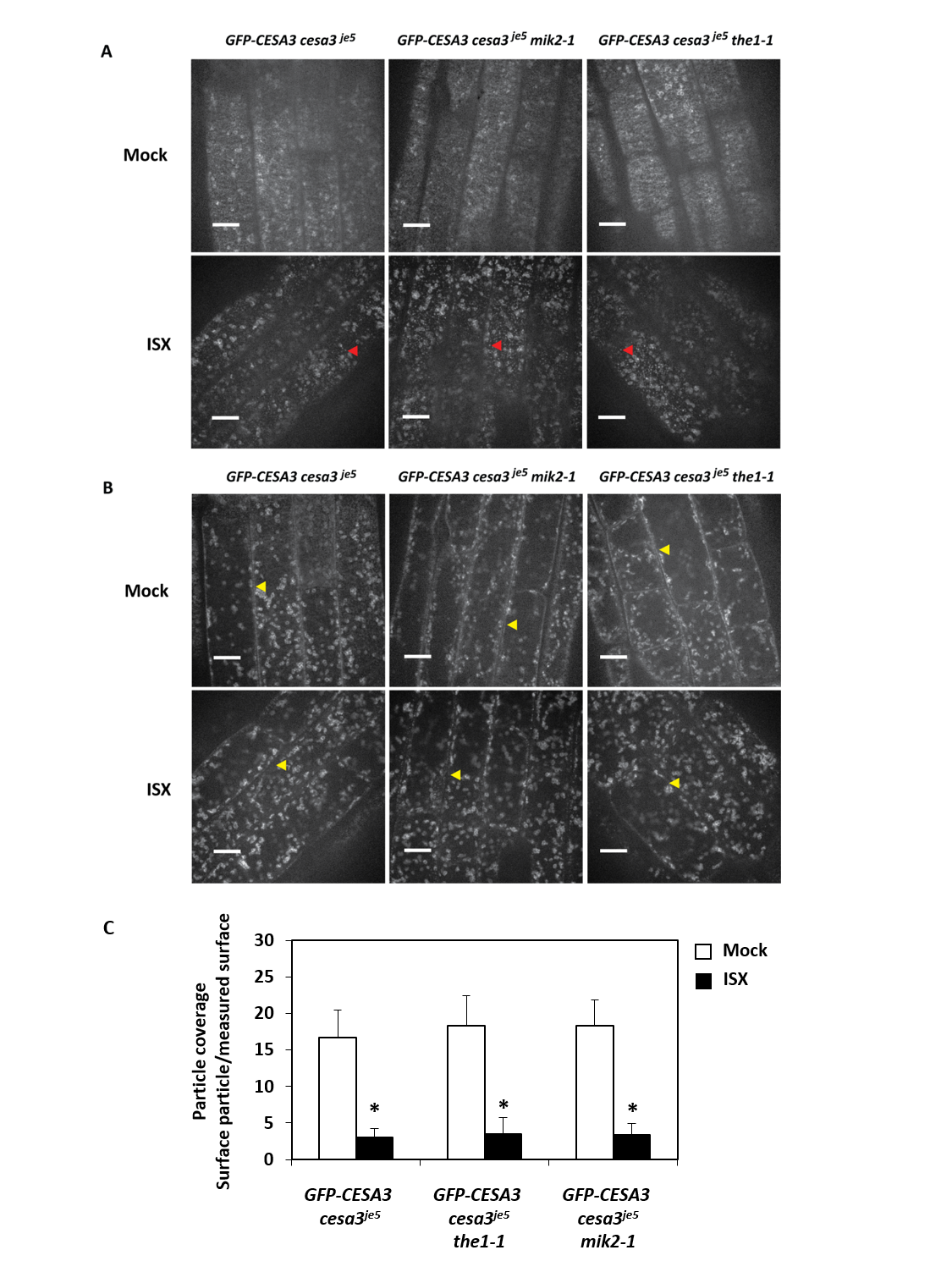

Supplement: S5 Fig — (A,B) Confocal images of GFP-CESA3 in cesa3je5, cesa3je5 mik2-1, or cesa3je5 the1-1 genetic background. Four-day-old Arabidopsis seedlings were mock treated or treated with 0.1 μM ISX for 2 h. Panel A displays the cell surface, while panel B displays a cross section through the cells. ISX treatment results in internalization of GFP-CESA3; GFP-CESA3 accumulates in microtubule-associated cellulose synthase compartments (MASCs) in the cell cortex. In panel A the red arrows indicate GFP-CESA3 in MASCs. In panel B the yellow arrows indicate the position of the plasma membrane, which is rich in GFP-CESA3 signal upon mock treatment and depleted of GFP-CESA3 after ISX treatment. The large circular fluorescent organelles are GFP-CESA3 signal in the Golgi apparatus. The size bars represent 10 μm. (C) Quantification of the surface particles depicted in (A). Asterisks indicate a statistically significant difference as determined by a two-tailed Student’s T-test (p < 0.05). Error bars represent the standard error of n = 80 measurements in 15 seedlings. The particle density analysis was performed as described [81]. (TIF) [file pgen.1006832.s005.tif]

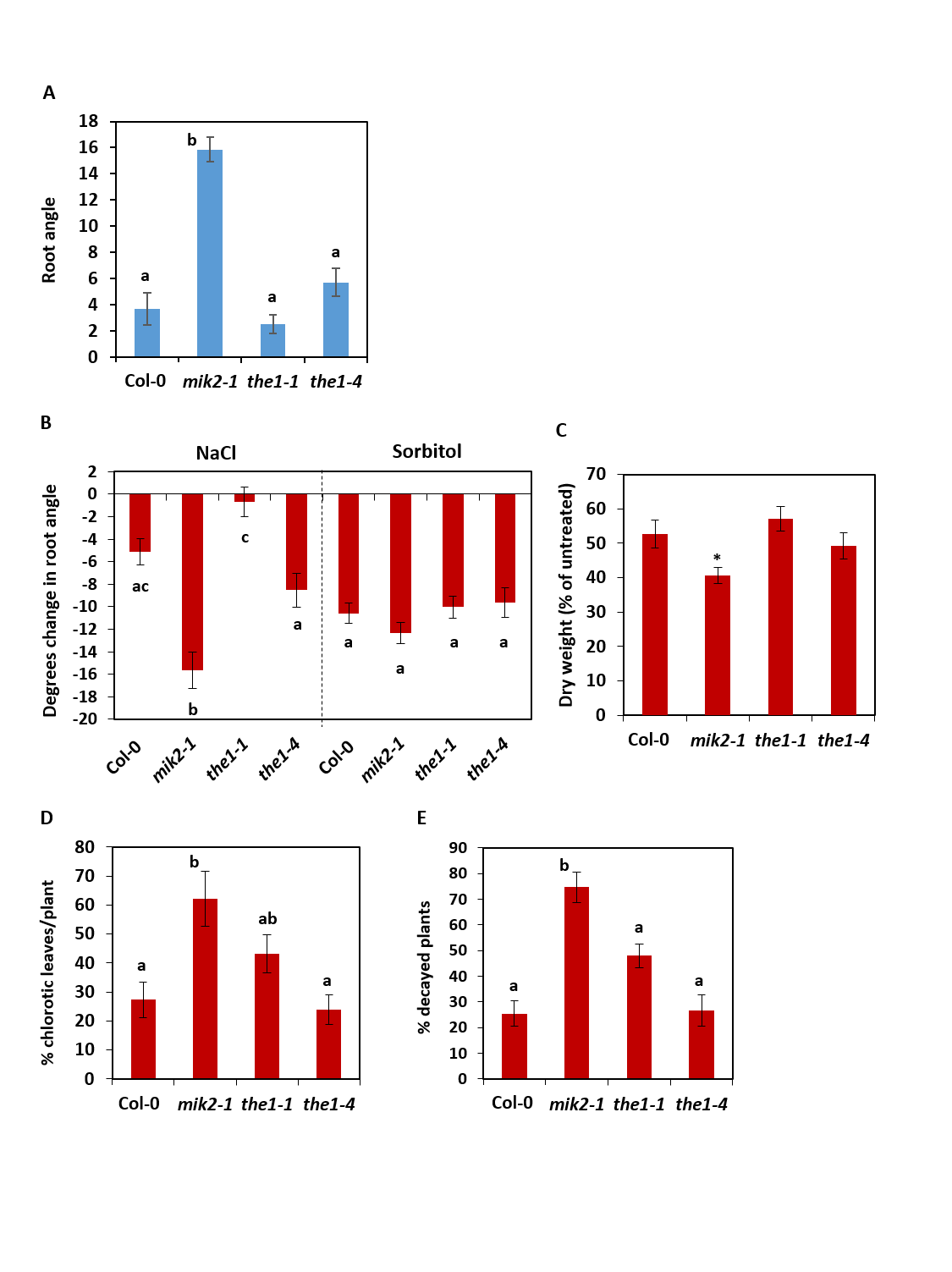

Supplement: S6 Fig — (A) Nine-day-old Arabidopsis seedlings grown in an upright position (under a 10° angle relative to the direction of gravity) on MS agar medium with 1% sucrose. Root angle relative to the vertical growth axis, and root length were quantified. Error bars represent standard error of n = 15 biological replicas. (B) Ten-day-old Arabidopsis seedlings were grown in an upright position on ½ MS agar medium without sucrose, supplemented with or without 75 mM NaCl or 150 mM sorbitol. Depicted is the change in the angle of the root after NaCl or sorbitol treatment compared to mock treatment; the negative value indicates a change to the right. Error bars represent standard error of n = 20 biological replicas. (C) Dry weight of NaCl-treated plants as percentage of the dry weight of untreated plants. Plants were treated as described in Fig 4. Error bars represent the standard error of n = 20 plants. An asterisk indicates a significant difference from Col-0 according to a linear mixed model (p < 0.05) (D,E) Percentage of chlorotic leaves per plant (D), and percentage of decayed plants (E) after infection of the roots with F. oxysporum isolate Fo5176. The experiment was performed as described in Fig 5. The bars represent the average of three independent experiments, each consisting of n = 20–40 plants per genotype. Error bars represent the standard error of n = 3 experiments. No disease symptoms were observed on mock-inoculated plants for any of the genotypes (n = 10). (A,B,D,E) Different letters indicate statistically significant differences between genotypes (ANOVA and Tukey HSD test (p < 0.05)). The experiments were repeated at least three times with similar results. (TIF) [file pgen.1006832.s006.tif]

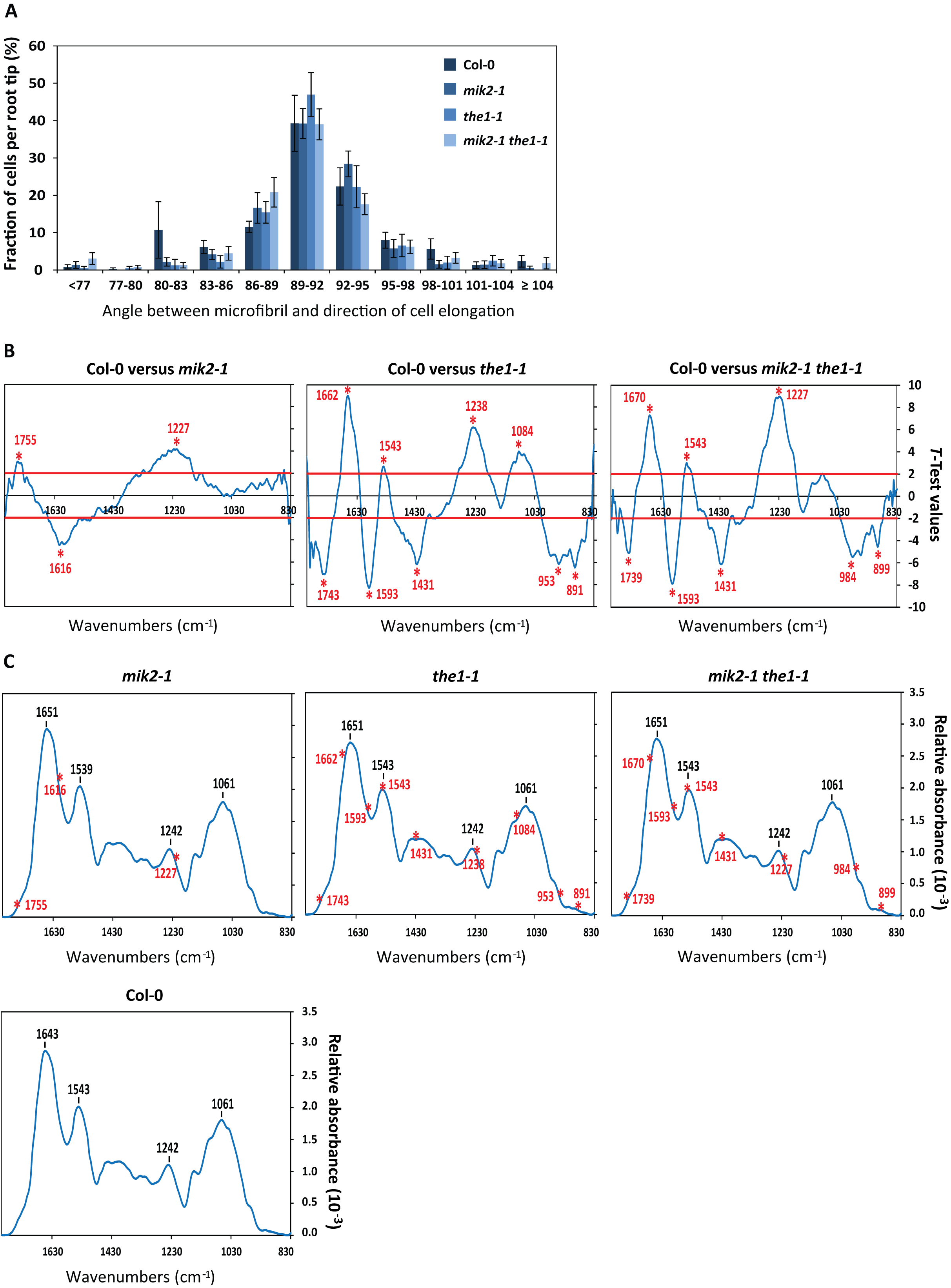

Supplement: S7 Fig — (A) Quantification of the orientation of cellulose microfibrils relative to the direction of cell elongation in root tips of 7-day-old Arabidopsis seedlings. Values of 3 independent experiments were combined. Error bars represent standard error of n = 10 roots. (B,C) FT-IR spectroscopy of root tips of 7 days-old Arabidopsis seedlings. Absorption spectra were collected along 800 μm of the root tip, spanning the elongation zone and the beginning of the differentiation zone. Absorption spectra of 4 independent experiments were combined and spectra of mik2-1, the1-1, and mik2-1 the1-1 were compared with Col-0. (B) T-test values for the indicated comparisons. T-test values above 2 or below -2 (marked by red lines) indicate statistically significant differences (p < 0.01). (C) Average absorbance spectra. Wavenumbers of the main 4 peaks are indicated in black. (B,C) Asterisks high-light points were mutants differ significantly from Col-0; corresponding wavenumbers are indicated in red. (TIF) [file pgen.1006832.s007.tif]

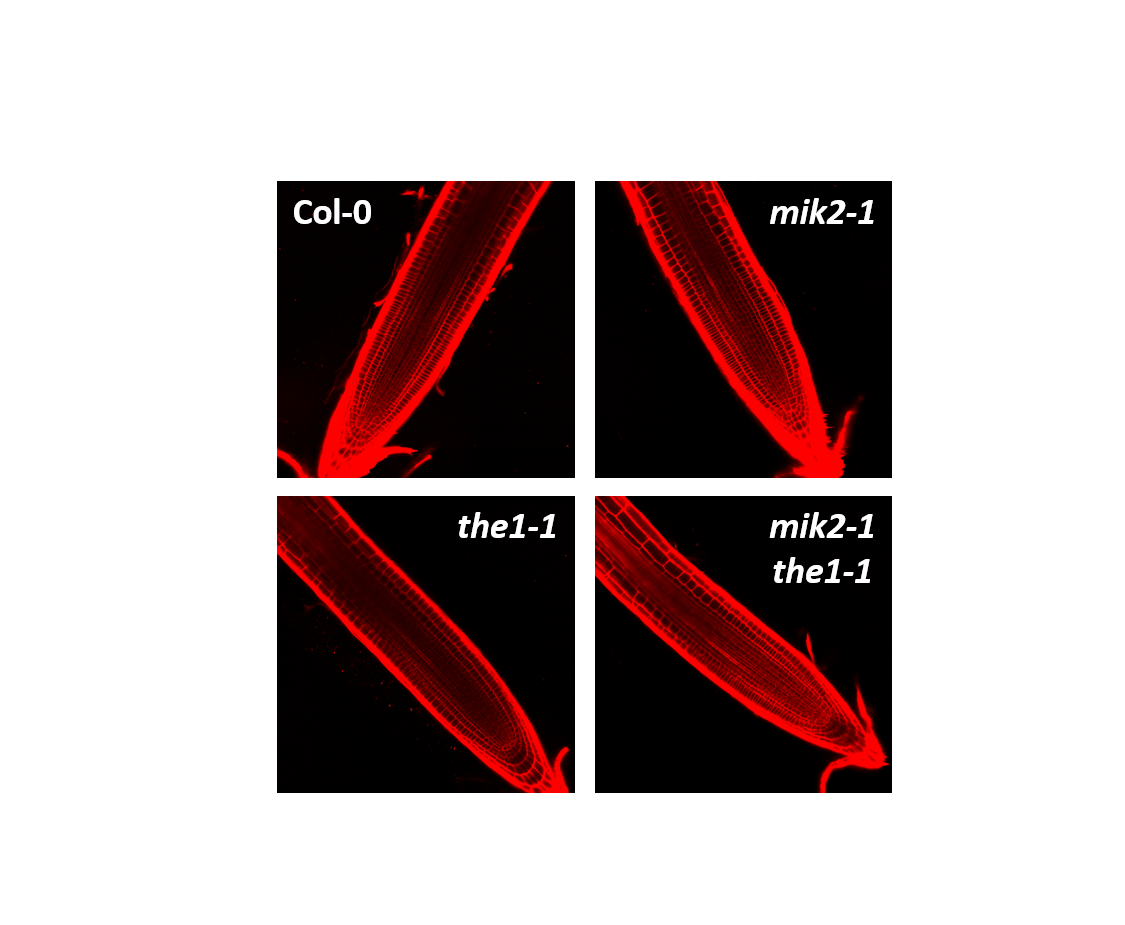

Supplement: S8 Fig — mik2-1, the1-1 and mik2-1 the1-1 mutants do not display any apparent defects in phloem continuity or root meristem morphology. (A) Confocal microscopy pictures of the root meristem of 7-day-old seedlings of the indicated genotypes stained with propidium iodide (red). Protophloem is visible as a bright, uninterrupted strand within the stele. (B) Cross sections of the root meristem of 5-day-old seedlings of the indicated genotypes, stained with toluidine blue. The number of cell files in the stele is quantified in (C) (n≥14; the mutant values are not significantly different from the Col-0 control [student’s t-test]). (TIF) [file pgen.1006832.s008.tif]

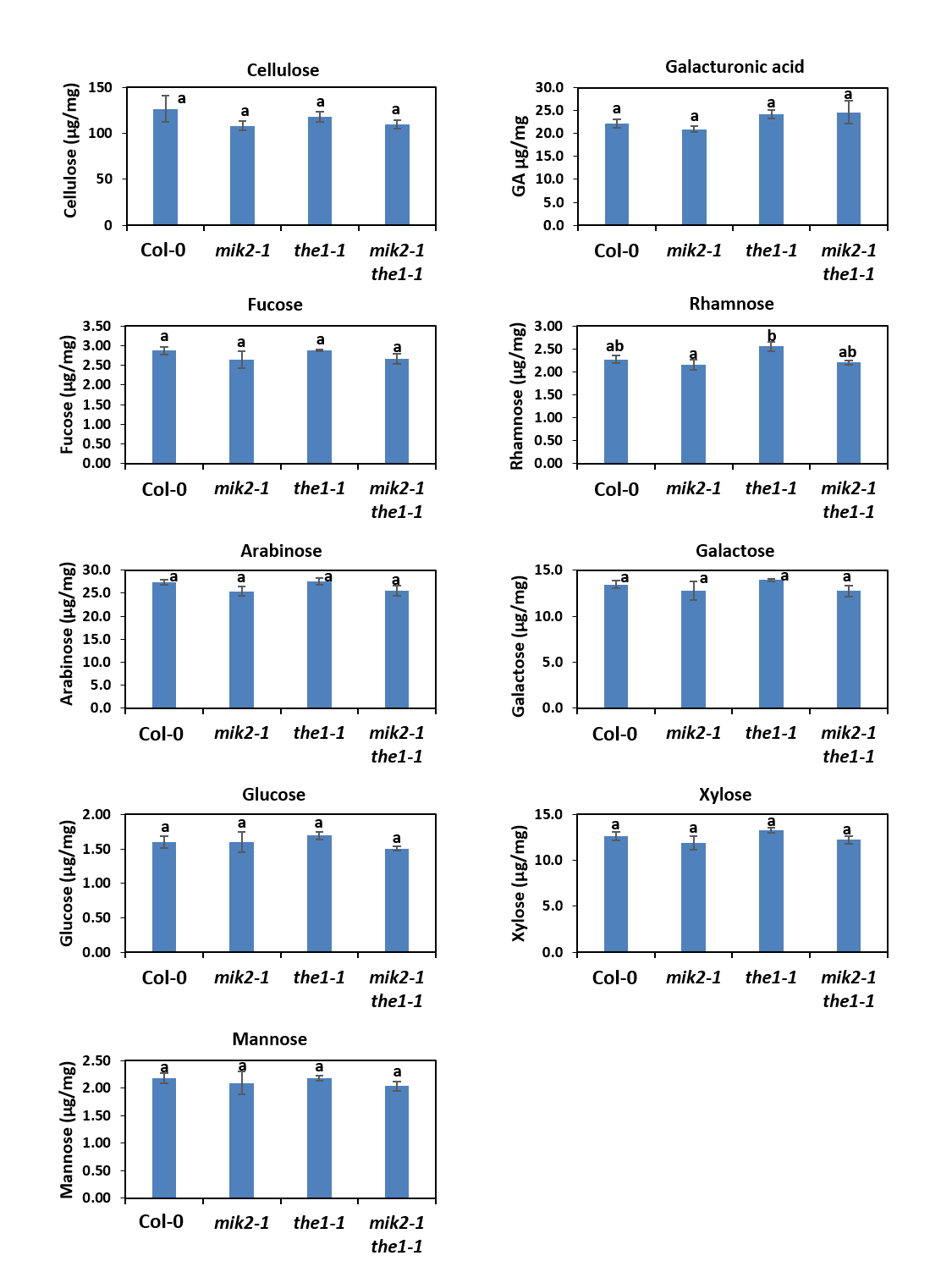

Supplement: S9 Fig — Levels of cellulose, pectin (galacturonic acid (GA)), and monosaccharides derived from hemi-cellulose or pectin, in roots of 7-day-old Arabidopsis seedlings. Values are expressed per mg root tissue. Depicted is the average of four independent experiments, and error bars represent standard error. Different letters indicate a statistically significant difference between genotypes (ANOVA followed by Tukey HSD test (p < 0.05)). (TIF) [file pgen.1006832.s009.tif]

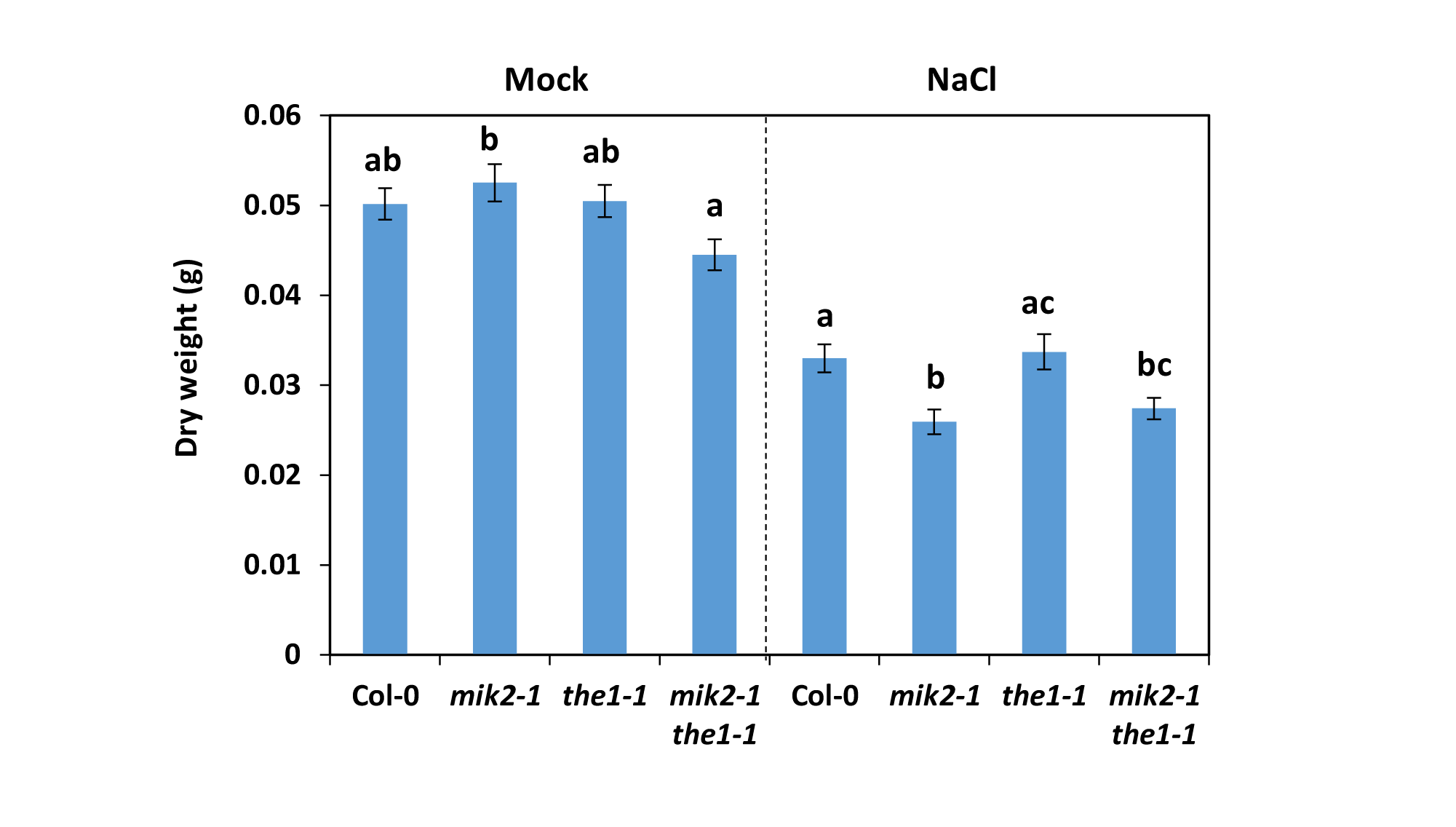

Supplement: S10 Fig — Dry weight of Arabidopsis plants treated with or without NaCl, as described in Fig 4B. Different letters indicate statistically significant differences between genotypes (Left panel: ANOVA and Holm-Sidak test (p < 0.05), right panel: Kruskal-Wallis ANOVA on ranks followed by Dunn’s multiple comparison procedures (p <0.05)). (TIF) [file pgen.1006832.s010.tif]

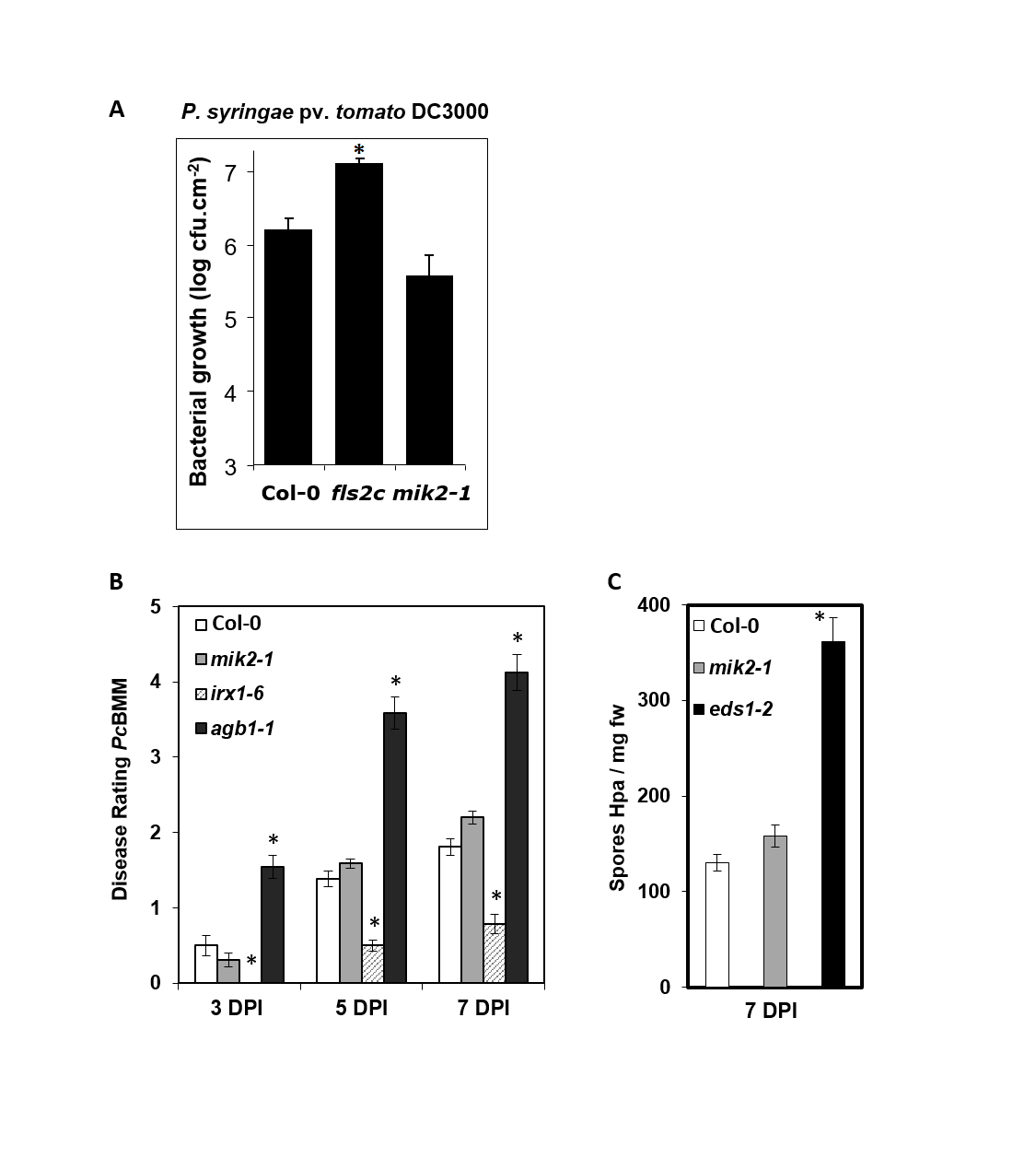

Supplement: S11 Fig — (A) Growth of Pseudomonas syringae pv. tomato DC3000 in Col-0 and mik2-1 mutant plants. The hypersusceptible mutant fls2c was included as a control. Plants were sprayed with a P. syringae bacterial suspension (OD600 = 0.02), and material was harvested two days later for quantification of bacterial growth. (B) Plant disease rating at different days post inoculation (dpi) with the necrotrophic fungus Plectosphaerella cucumerina BMM (PcBMM). Three-week-old Arabidopsis Col-0 plants, the mik2-1 mutant, and the irx1-6 and agb1-1 mutants, included as resistant and hypersusceptible controls, respectively, were inoculated with 4 x 10^6^ spores/mL of PcBMM. Quantification of fungal growth was estimated by visual evaluation of the plant disease symptoms (from 0 to 5) and average disease rating was determined. Values are means ± standard deviation (n = 10). (C) Resistance to the biotrophic pathogen Hyaloperonospora arabidospsidis (Hpa). Two-week-old plants of the indicated genotypes and the Hpa hypersusceptible eds1-2 mutant were inoculated with 5 x 10^4^ spores/mL Hpa. Fungal growth in leaves was determined 7 dpi by measuring Hpa sporulation (Hpa spores/mg plant fresh weight (fw)). Values are means ± standard deviation (n = 10). (A-C) The experiments were repeated at least three times with similar results. Statistically significant values (*) that differ from those of wild-type plants were determined by Student’s T-test (p < 0.05). (TIF) [file pgen.1006832.s011.tif]

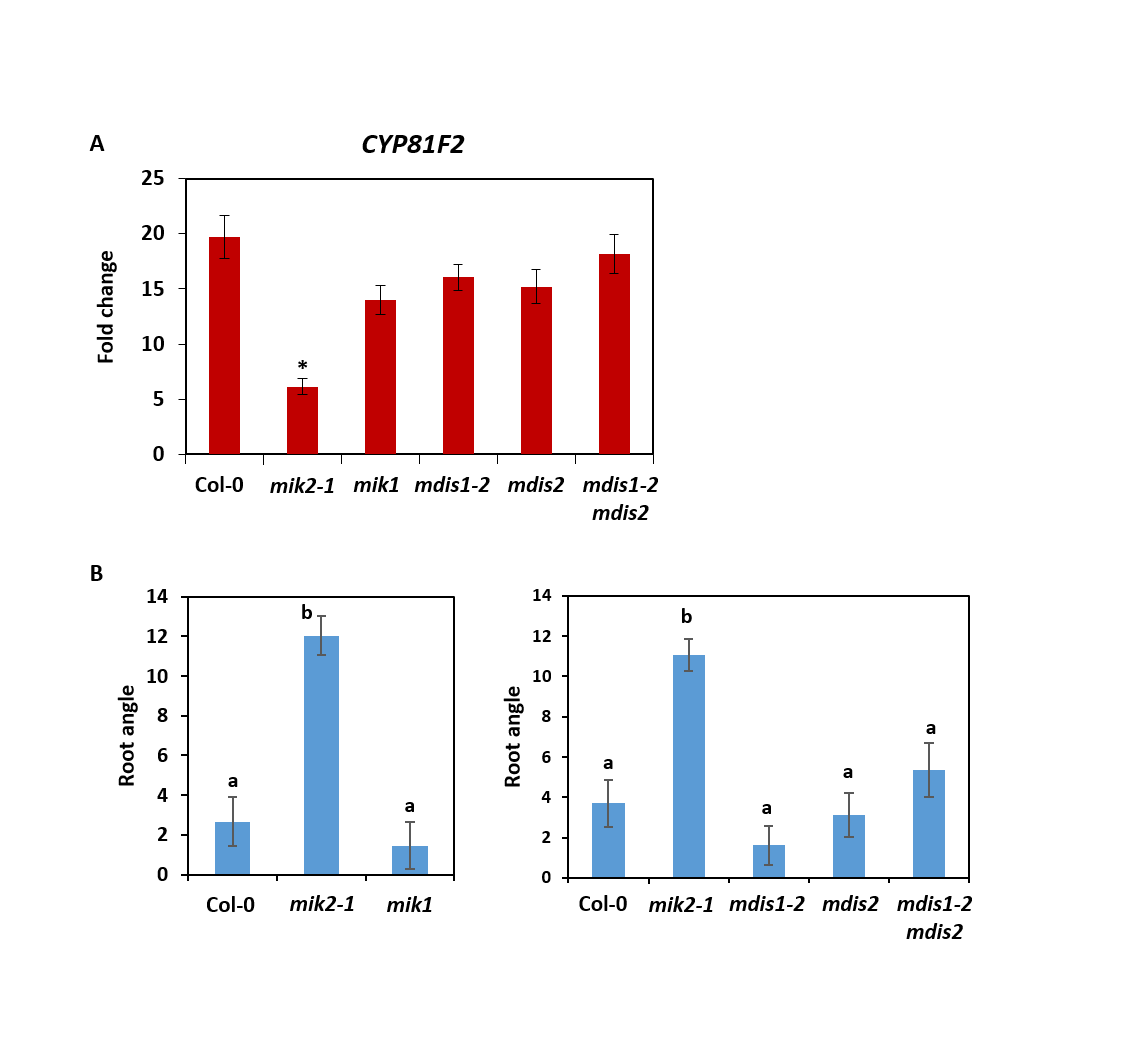

Supplement: S12 Fig — (A) Immune marker gene expression in 13-day-old Arabidopsis seedlings determined by qRT-PCR. Seedlings were mock treated, or treated with 0.6 μM ISX for 9 h. Expression of the immune marker gene CYP81F2 was normalized relative to U-box expression values. Depicted is the fold change in expression relative to mock treatment. Error bars represent standard error of three technical replicas. The asterisk indicates a statistically significant difference relative to Col-0, as determined by a two-tailed Student’s T-test (p < 0.05). (B) Nine-day-old Arabidopsis seedlings grown in an upright position (under a 10° angle relative to the direction of gravity) on MS agar medium with 1% sucrose. Root angle relative to the vertical growth axis was quantified. Error bars represent standard error of n = 15 biological replicas. Different letters indicate statistically significant differences between genotypes (ANOVA and Tukey HSD test (p < 0.05)). (A,B) The experiments were repeated at least three times with similar results. (TIF) [file pgen.1006832.s012.tif]
